# Cotargeting of BTK and MALT1 overcomes resistance to BTK inhibitors in mantle cell lymphoma

**DOI:** 10.1172/JCI165694

**Published:** 2023-02-01

**Authors:** Vivian Changying Jiang, Yang Liu, Junwei Lian, Shengjian Huang, Alexa Jordan, Qingsong Cai, Ruitao Lin, Fangfang Yan, Joseph McIntosh, Yijing Li, Yuxuan Che, Zhihong Chen, Jovanny Vargas, Maria Badillo, John Nelson Bigcal, Heng-Huan Lee, Wei Wang, Yixin Yao, Lei Nie, Christopher R. Flowers, Michael Wang

**Affiliations:** 1 Department of Lymphoma and Myeloma and; 2 Department of Biostatistics, The University of Texas MD Anderson Cancer Center, Houston, Texas, USA.; 3 Center for Precision Health, School of Biomedical Informatics, The University of Texas Health Science Center at Houston, Houston, Texas, USA.; 4 Department of Stem Cell Transplantation and Cellular Therapy, The University of Texas MD Anderson Cancer Center, Houston, Texas, USA.

**Keywords:** Hematology, Therapeutics, Drug therapy, Lymphomas, Signal transduction

## Abstract

Bruton’s tyrosine kinase (BTK) is a proven target in mantle cell lymphoma (MCL), an aggressive subtype of non-Hodgkin lymphoma. However, resistance to BTK inhibitors is a major clinical challenge. We here report that *MALT1* is one of the top overexpressed genes in ibrutinib-resistant MCL cells, while expression of *CARD11*, which is upstream of MALT1, is decreased. *MALT1* genetic knockout or inhibition produced dramatic defects in MCL cell growth regardless of ibrutinib sensitivity. Conversely, *CARD11*-knockout cells showed antitumor effects only in ibrutinib-sensitive cells, suggesting that MALT1 overexpression could drive ibrutinib resistance via bypassing BTK/CARD11 signaling. Additionally, *BTK* knockdown and *MALT1* knockout markedly impaired MCL tumor migration and dissemination, and MALT1 pharmacological inhibition decreased MCL cell viability, adhesion, and migration by suppressing NF-κB, PI3K/AKT/mTOR, and integrin signaling. Importantly, cotargeting MALT1 with safimaltib and BTK with pirtobrutinib induced potent anti-MCL activity in ibrutinib-resistant MCL cell lines and patient-derived xenografts. Therefore, we conclude that MALT1 overexpression associates with resistance to BTK inhibitors in MCL, targeting abnormal MALT1 activity could be a promising therapeutic strategy to overcome BTK inhibitor resistance, and cotargeting of MALT1 and BTK should improve MCL treatment efficacy and durability as well as patient outcomes.

## Graphical abstract

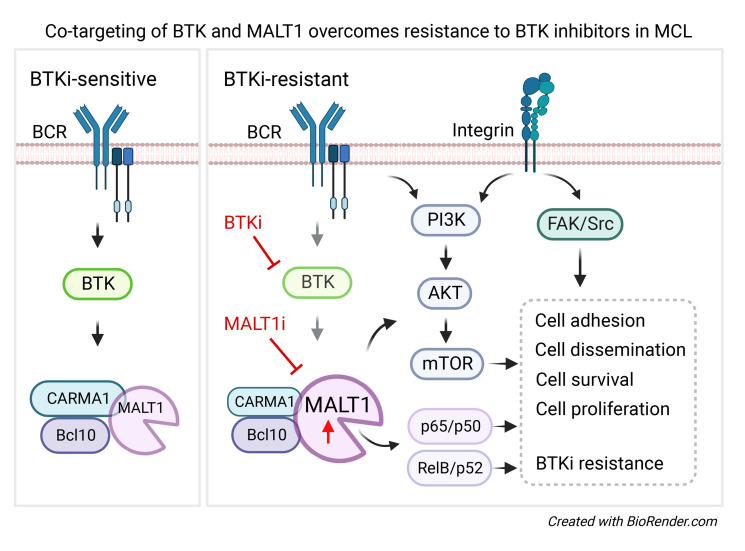

## Introduction

Mantle cell lymphoma (MCL) is an incurable subtype of non-Hodgkin lymphoma with aberrant activation of the B cell receptor (BCR) pathway. Upon BCR stimulation, Bruton’s tyrosine kinase (BTK) is activated, which in turn regulates the downstream NF-κB pathway and the PI3K/AKT/mTOR pathway (1–3). MALT1 is an essential regulator of BCR-activated NF-κB signaling and serves 2 intrinsic functions, as a scaffold protein and as the sole human paracaspase (4–7). As a scaffold protein, MALT1 binds to CARD11 and BCL10 to form the CARD11-BCL10-MALT1 complex, which activates IKK and NF-κB signaling (8–10). As a paracaspase, MALT1 cleaves substrates that include itself, BCL10, RelB, A20, and CYLD to dramatically upregulate NF-κB activation (11–18). MALT1 also regulates nonclassical NF-κB signaling by physically interacting with TRAF3 (19). However, the roles of MALT1 in lymphomagenesis and resultant drug resistance are poorly understood.

Pharmacological BTK inhibitors (BTKis) like ibrutinib (IBN, covalent) and pirtobrutinib (PBN, noncovalent) have been proven effective in MCL treatment. However, MCL patients frequently relapse from these treatments (20–25). Nevertheless, PBN was recently shown to overcome resistance to covalent BTKis in 52% of MCL patients (24), indicating that BTK is still targetable in this cohort. Recent studies have shown that noncanonical NF-κB signaling is associated with IBN resistance (26), and PI3K/AKT/mTOR and integrin signaling also confer tumor microenvironment–driven (TME-driven) IBN resistance in MCL (27). However, the role of MALT1 in BTKi resistance is not well defined, and novel means to overcome BTKi resistance are needed.

In this study, we found that MALT1 was overexpressed in IBN-resistant (IBN-R) MCL cells. Genetic knockout (KO) or pharmacological inhibition revealed that MALT1, but not CARD11, drives BTKi resistance via bypassing upstream BTK/CARD11 signaling. Unbiased transcriptome and protein profiling revealed that MALT1 inhibition potently suppressed NF-κB, PI3K/AKT/mTOR, and integrin signaling, and diminished MCL cell proliferation and dissemination. Importantly, combinatorial treatment with MALT1 inhibitors (e.g., MI-2 and safimaltib) and BTKis (e.g., IBN and PBN) produced potent anti-MCL activity in both IBN-R MCL cell lines and patient-derived xenograft (PDX) models. Thus, we envision that cotargeting of MALT1 and BTK could be a promising therapeutic strategy to overcome BTKi resistance in MCL.

## Results

### MALT1 is overexpressed in IBN-R MCL cells.

**Figure 1:**
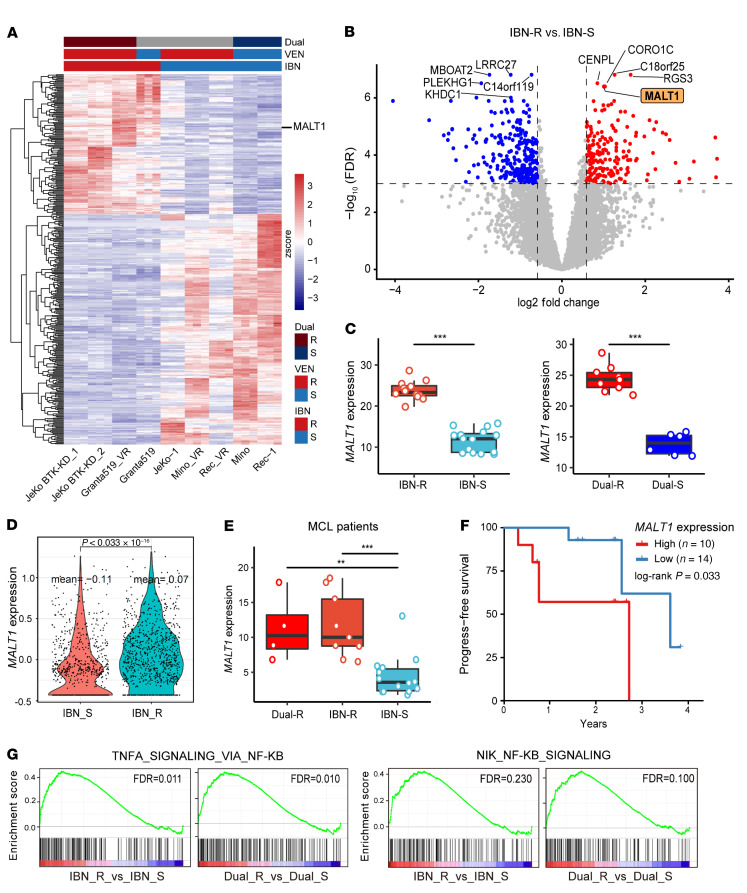
MALT1 is overexpressed in ibrutinib-resistant MCL cell lines and primary MCL cells. (**A**) Heatmap with *MALT1* highlighted in the right as one of the top DEGs in IBN-R MCL cells compared with IBN-S cells. (**B**) Volcano plot shows *MALT1* was upregulated in the IBN-R group. (**C**) *MALT1* mRNA expression in IBN-R versus IBN-S and Dual-R versus Dual-S groups. (**D**) Violin plot shows *MALT1* mRNA expression in IBN-R (*n* = 17) versus IBN-S (*n* = 4) MCL cells at single-cell resolution determined by single-cell RNA sequencing. Statistical significance was calculated using Wilcoxon’s rank-sum test. (**E**) *MALT1* mRNA expression determined by qPCR in IBN-R (*n* = 9), Dual-R (*n* = 4), and IBN-S (*n* = 13) cells. Statistical significance was determined based on the adjusted *P* values using Dunnett’s approach. **P < 0.01; ***P < 0.001. (**F**) High *MALT1* mRNA expression correlated with progress-free survival in MCL patients. The log-rank test was used to assess the statistical significance of progression-free survival. (**G**) GSEA identifies NF-κB signaling pathways as top cancer hallmarks that were upregulated in IBN-R cells compared with IBN-S cells. FDRs were generated using the Benjamini-Hochberg method. Box-and-whisker plots in **C** and **E** show the median ± 1 quartile, with whiskers extending from the hinge to the smallest and largest values within 1.5 × (interquartile range) from the box boundaries. The values beyond the ends of the whiskers are outliers. All other data represent the mean ± SD.

BTKis like ibrutinib and BCL2 inhibitors like venetoclax (VEN) have been demonstrated to be highly efficacious in treating patients with MCL. However, the development of single or dual resistance to targeted therapy is common. To investigate the potential mechanism underlying this resistance, we performed whole-transcriptome profiling of 9 MCL cell lines that had varying degrees of targeted agent sensitivity (see Methods) (Supplemental Figure 1A; supplemental material available online with this article; https://doi.org/10.1172/JCI165694DS1). *MALT1* was among the top differentially expressed genes (DEGs) across the genome-wide transcriptome and among all NF-κB signaling genes, and it was more highly expressed in IBN-R than IBN-sensitive (IBN-S) cells (*P* < 0.001) (Figure 1, A–C, and Supplemental Figure 1, B and C). *MALT1* expression was also significantly higher in cells resistant to both IBN and VEN (Dual-R cells) than in cells sensitive to both drugs (Dual-S cells) (*P* < 0.001) (Figure 1C). Interestingly, expression of *CARD11*, which encodes the upstream binding partner of MALT1, was lower in IBN-R MCL cells (Supplemental Figure 1, B and C).

Elevated *MALT1* expression was also observed in IBN-R MCL cells (*P* < 2.22 × 10^–16^) compared with IBN-S MCL cells in primary patient samples, based on our single-cell RNA sequencing analysis (Figure 1D). Furthermore, quantitative PCR revealed that *MALT1* expression was also higher in primary MCL cells compared with healthy PBMCs (*P* < 0.05) (Supplemental Figure 1D). Consistent with the whole-transcriptomic analysis, IBN-R and Dual-R MCL patient samples expressed significantly higher levels of *MALT1* compared with IBN-S MCL patient samples (*P* < 0.001 and *P* < 0.01, respectively) (Figure 1E). High *MALT1* expression was associated with poor clinical outcomes (*P* < 0.05) (Figure 1F) as it also was for 2 similarly defined patient cohorts described by others (*P* < 0.05) (Supplemental Figure 1, E and F) (28, 29).

MALT1 is crucial in mediating NF-κB and other signaling pathways (8–10, 19). Gene set enrichment analysis (GSEA) revealed that multiple cancer signaling hallmarks, including MYC targets, NF-κB signaling, and G_2_/M checkpoint, were associated with IBN-R and Dual-R MCL cells (Supplemental Figure 1G). Importantly, deeper analysis showed that both canonical and noncanonical NF-κB signaling were upregulated in IBN-R and Dual-R cells at the transcriptional level (Figure 1G). This was further confirmed by the activation of NF-κB family members in IBN-R cells compared with IBN-S cells (Supplemental Figure 1H), suggesting upregulated NF-κB signaling associated with poor MCL patient survival.

### MALT1 acts as an oncogenic driver of IBN resistance in MCL cells.

**Figure 2:**
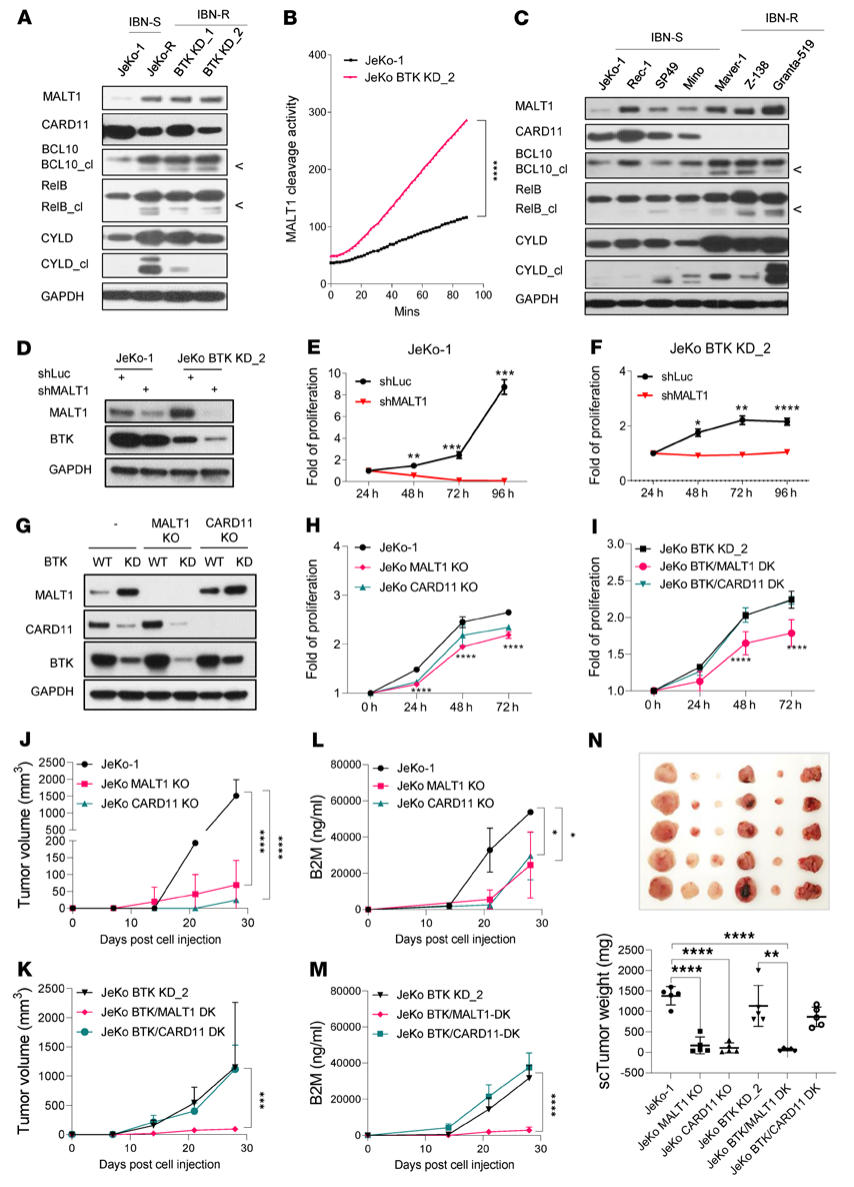
MALT1 acts as an oncogenic tumor driver in ibrutinib-resistant MCL cells. (**A**) Expression of MALT1, CARD11, and BCL10, and cleavage (cl) of MALT1 substrates, in JeKo-1 and JeKo-1–derived cell lines. (**B**) Endogenous MALT1 cleavage activity detected in JeKo-1 and JeKo BTK KD-2 cells. Statistical significance was determined based on the *F* test of the slope from the linear regression model. (**C**) Expression of MALT1, CARD11, BCL10, and cleavage of MALT1 substrates in 7 additional MCL cell lines. (**D**) Expression of MALT1 in JeKo-1 cells and JeKo BTK KD-2 cells, with or without *MALT1* or *CARD11* KD by shRNA. (**E** and **F**) *MALT1* KD resulted in cell proliferation inhibition in JeKo-1 (**E**) and JeKo BTK KD-2 (**F**) cells. (**G**) Expression of MALT1, CARD11, and BTK in JeKo-1 and JeKo BTK KD-2 cells with or without *MALT1* KO, *CARD11* KO, or *BTK* KD. (**H**) *MALT1* KO or *CARD11* KO led to decreased cell proliferation in JeKo-1 cells. (**I**) *MALT1* KO, but not *CARD11* KO, led to diminished cell proliferation in JeKo BTK KD-2 cells. (**J**–**N**) CDX models were established by subcutaneous injection of 5 × 10^6^ JeKo-1 cells and JeKo BTK KD-2 cells, with or without *MALT1* or *CARD11* KO (*n* = 5 per group). Tumor size was monitored weekly (**J** and **K**). Serum B2M level (serving as a systematic tumor load indicator) was measured by ELISA (**L** and **M**). At the end of experiments, subcutaneous tumors were dissected, weighed, and imaged (**N**). Two-way ANOVA was used in **E** and **F** and **H**–**M** to assess the effect of 2 factors (i.e., time and cell line) and 1-way ANOVA was used in **N**. Statistical significance was determined based on the adjusted *P* values using Šídák’s method. Data represent mean ± SD. *P < 0.05; **P < 0.01; ***P < 0.001; ****P < 0.0001.

To investigate how MALT1 overexpression may confer IBN resistance, we first assessed MALT1 protein levels in JeKo BTK KD-1 and -2 cells with intrinsic IBN resistance and in JeKo-R cells with acquired IBN resistance. These IBN-R cells expressed much higher levels of MALT1 protein, even as CARD11 protein was expressed at notably reduced levels (Figure 2A). To determine whether MALT1 overexpression was correlated with increased paracaspase activity, we examined the cleavage of MALT1 substrates. Higher MALT1 expression indeed correlated positively with the increased cleavage of its substrates (Figure 2A). MALT1 endogenous cleavage activity in JeKo BTK KD-2 cells was further confirmed to be significantly higher than that observed in JeKo-1 cells (*P* < 0.0001) (Figure 2B).

MALT1 overexpression and its heightened paracaspase activity were further validated in MCL cell lines having primary IBN resistance (Figure 2C) and in primary patient MCL cells (Supplemental Figure 2A). Transient knockdown (KD) of *MALT1* expression by MALT1 shRNA (30) resulted in significant cell proliferation perturbation in vitro in all MCL cell lines tested at 24 (*P* < 0.05), 48 (*P* < 0.05), and 72 (*P* < 0.001) hours (Figure 2, D–F, and Supplemental Figure 2, B–D). Further, ectopic expression of *MALT1* induced by doxycycline in JeKo-MALT1 cells promoted cell proliferation at days 3 to 5 (*P* < 0.01) compared with parental JeKo-1 cells (Supplemental Figure 2, E and F). This suggested that MALT1, as a key BTK downstream signaling molecule, was critical for MCL cell proliferation.

We next used CRISPR/Cas9 technology to generate stable cell lines from JeKo-1 and JeKo BTK KD-2 cells with KO of *MALT1* or *CARD11*. No MALT1 or CARD11 protein was detected in the resulting cell lines (Figure 2G). Consistent with the transient KD assessment, stable KO of *MALT1* led to significant suppression of cell proliferation in both JeKo-1 and JeKo BTK KD-2 cells (*P* < 0.0001) (Figure 2, H and I). In contrast, stable KO of *CARD11* resulted in notable inhibition of cell proliferation in IBN-S JeKo-1 cells but not IBN-R JeKo BTK KD-2 cells (Figure 2, H and I).

We next established subcutaneous cell line–derived xenograft (CDX) models in immunodeficient NOD.Cg-*Prkdc^scid^* *Il2rg^tm1Wjl^*/SzJ (NSG) mice. Consistent with the in vitro cell proliferation assays (Figure 2, H and I), *MALT1* KO greatly suppressed tumor growth and serum levels of β-2-microglobulin (B2M), a systematic indicator of tumor load, in both JeKo-1 (*P* < 0.0001 and *P* < 0.05, respectively) and JeKo BTK KD-2 CDX (*P* < 0.001 and *P* < 0.0001, respectively) models, while *CARD11* KO suppressed tumor growth and serum levels of B2M in only the JeKo-1 CDX model 1 (*P* < 0.0001 and *P* < 0.05, respectively) (Figure 2, J–N). These data demonstrate that MALT1 is crucial in driving MCL tumorigenesis and IBN resistance via a compensatory mechanism that appears to bypass upstream BTK/CARD11 signaling.

### MI-2 blocks MALT1’s paracaspase activity and MCL cell proliferation by suppressing NF-κB signaling.

**Figure 3:**
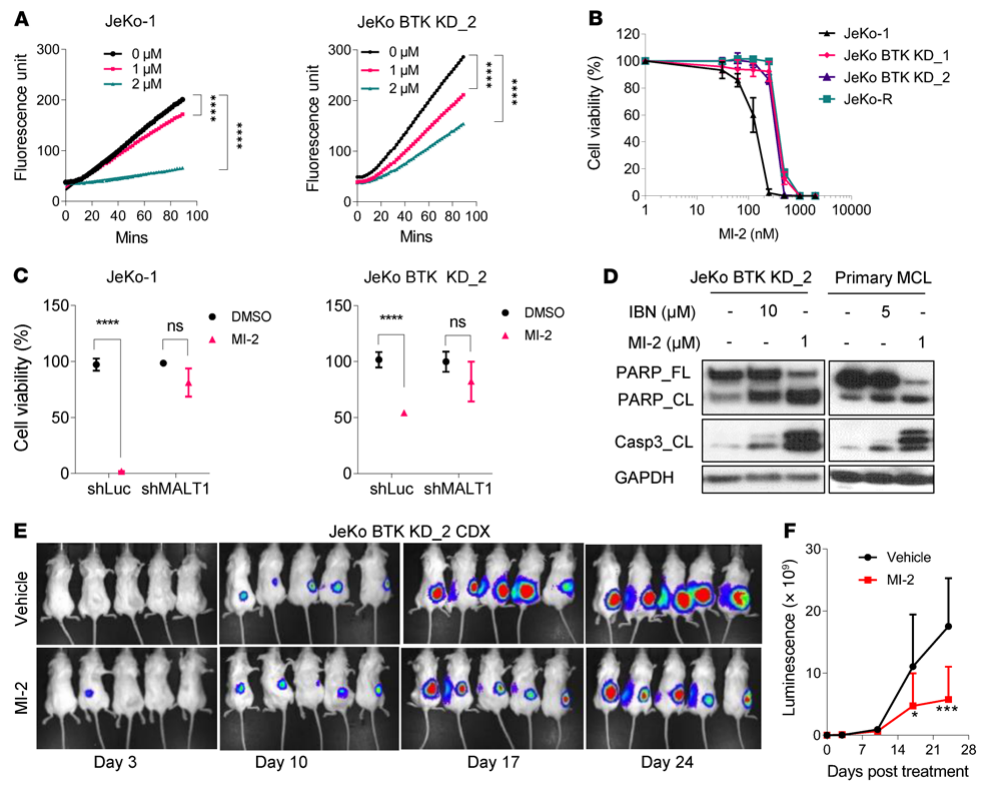
MALT1 inhibition by MI-2 decreases MALT1 paracaspase activity and suppresses proliferation in MCL cells. (**A**) Endogenous MALT1 cleavage activity detected in JeKo-1 cells and JeKo BTK KD-2 cells upon MALT1 inhibition by MI-2 at the indicated concentrations and treatment times. Each treatment for the indicated cell lines was set up in triplicate. Statistical significance was determined based on the *F* test of the slope from the linear regression model and multiple comparison was adjusted using Šídák’s approach. (**B**) MALT1 inhibitor MI-2 potently inhibited viability in JeKo-1, JeKo-R, and JeKo BTK KD-1 and -2 cells. (**C**) MI-2 effectively inhibited viability in JeKo-1 cells and JeKo BTK KD-2 cells, but not their counterparts with stable MALT1 KD. Error bars were generated from at least 3 independent replicates (**B** and **C**). (**D**) MI-2 induced cleavage (CL) of full-length PARP (PARP_FL) and caspase 3 in JeKo BTK KD-2 cells and primary patient cells. (**E** and **F**) NSG mice bearing luciferase-expressing JeKo BTK KD-2–derived subcutaneous xenografts were treated with vehicle (*n* = 5) or MI-2 (*n* = 5) at 25 mg/kg daily via intraperitoneal injection for 24 days. Tumor growth was monitored by live animal luminescence imaging (**E**) and the luciferase flux was plotted (**F**). Two-way ANOVA was used in **C** and **F**, and statistical significance was determined based on the adjusted *P* values using Šídák’s method. *P < 0.05; ***P < 0.001; ****P < 0.0001.

To assess the potential role of MALT1’s paracaspase activity, we treated MCL cells with MI-2 (31). A 6-hour MI-2 treatment suppressed endogenous MALT1 cleavage activity in a dose-dependent manner in JeKo-1 (*P* < 0.0001), JeKo BTK KD-2 (*P* < 0.0001), and Z138 (*P* < 0.0001) cells (Figure 3A and Supplemental Figure 3A). A 72-hour treatment with MI-2 produced a potent loss of viability in JeKo-1– and JeKo-derived resistant cells as well as other cell lines with half-maximal inhibitory concentrations (IC_50_) in the nanomolar range (Figure 3B and Supplemental Figure 3, B and C). After 24 hours of treatment, MI-2 also effectively inhibited cell viability of primary MCL patient cells resistant to IBN (Supplemental Figure 3D). This activity was lost when MALT1 expression was depleted by shRNA in JeKo-1 and JeKo BTK KD-2 cells (Figure 3C). MI-2 also induced robust apoptosis in MCL cells regardless of their IBN sensitivity (*P* < 0.001) (Supplemental Figure 3E), which was accompanied by cleavage of poly(ADP-ribose) polymerase (PARP) and caspase 3 in JeKo BTK KD-2 and primary MCL patient cells (Figure 3D). To further assess the potency of MI-2 against MCL in vivo, we engineered JeKo BTK KD-2 cells to stably express the luciferase reporter gene and then established JeKo BTK KD-2 CDX models. MI-2 treatment (25 mg/kg, daily) significantly inhibited tumor growth in these IBN-R CDX models (*P* < 0.001) (Figure 3, E and F).

**Figure 4:**
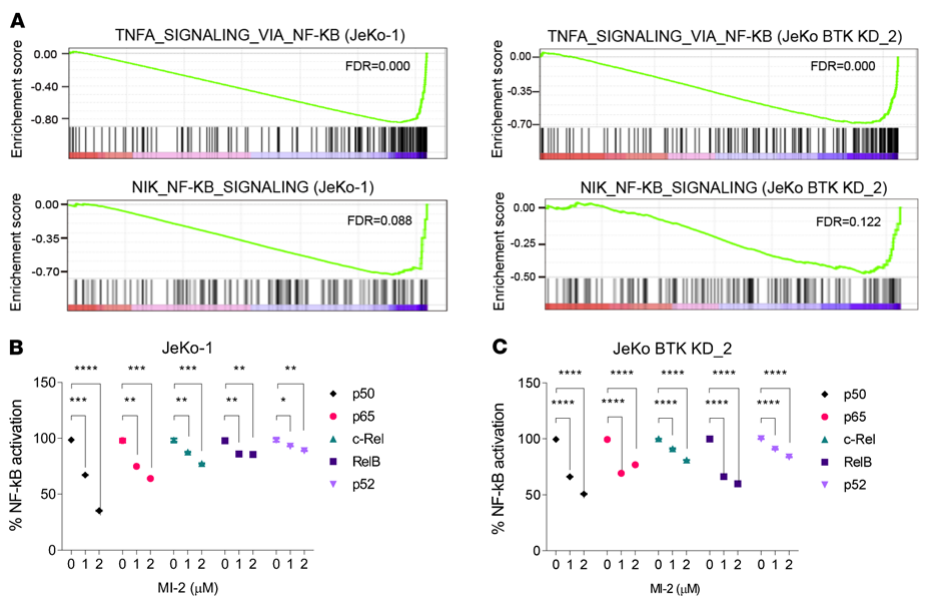
MALT1 inhibition by MI-2 suppresses NF-κB signaling in MCL cells. (**A**) The enrichment score of TNF-α signaling via NF-κB (upper panels) and NF-κB–inducing kinase (NIK) NF-κB signaling (bottom panels) in JeKo-1 (left panels) and JeKo BTK KD-2 (right panels) cells. (**B** and **C**) Activity of all 5 NF-κB family members was reduced upon MALT1 inhibition by MI-2 in JeKo-1 (**B**) and JeKo BTK KD-2 (**C**) cells. Error bars were generated from 3 independent replicates (**B** and **C**). Two-way ANOVA was used in **B** and **C**, and statistical significance was determined based on the adjusted *P* values using Šídák’s method. Data represent mean ± SD. *P < 0.05, **P < 0.01, ***P < 0.001, ****P < 0.0001.

Both canonical and noncanonical NF-κB signaling pathways were upregulated in IBN-R cells (Figure 1G), suggesting that MALT1 overexpression may be the key factor conferring their constitutive activation. To address this, we employed unbiased whole-transcriptome GSEA profiling of JeKo-1 and JeKo BTK KD-2 cells treated with MI-2 or vehicle (dimethyl sulfoxide, DMSO) for 6 hours. We identified NF-κB signaling and related inflammatory responses as the top downregulated pathways upon MALT1 inhibition (Supplemental Figure 3F). Our analysis also revealed that MALT1 inhibition suppressed both canonical and noncanonical NF-κB signaling in these cells (Figure 4A). This was further validated by assays assessing p65, p50, c-Rel, RelB, and p52 activity in JeKo-1 (*P* < 0.05) and JeKo BTK KD-2 (*P* < 0.0001) cells (Figure 4, B and C). In the IBN-S cells, canonical NF-κB family members were suppressed to a greater extent upon MALT1 inhibition compared with the noncanonical family members. Conversely, in the JeKo BTK KD-2 cells, both the canonical and noncanonical NF-κB family members were markedly inhibited.

Excess ROS production can trigger apoptosis (32), and NF-κB activation attenuates ROS production to promote cell survival (33). MALT1 inhibition triggered upregulation of the ROS pathway (*P* < 0.05) and ROS production (*P* < 0.05) and loss of mitochondrial membrane potential (ΔΨ_m_) (*P* < 0.0001) (Supplemental Figure 3, G and H), suggesting a role of mitochondrial dysfunction in the observed MI-2–induced ROS production. Together, these data suggest that targeting MALT1 paracaspase activity with MI-2 is effective in overcoming IBN resistance in MCL via modulating NF-κB activity and the ROS pathway.

### MALT1 and BTK are required for MCL cell dissemination in vivo.

Once disseminated, MCL can involve one or more lymph nodes, peripheral blood (PB), bone marrow (BM), spleen, liver, gastrointestinal tract, and even the central nervous system (34), as can be recapitulated using CDX or PDX xenografts (35). Further, MALT1-dependent cleavage of BCL10 reportedly controls integrin-dependent adhesion of T cells and MALT lymphomas (16, 36). Therefore, we speculated that MALT1 may mediate cell adhesion, and potentially MCL dissemination, via interaction between MCL cells and the TME.

**Figure 5:**
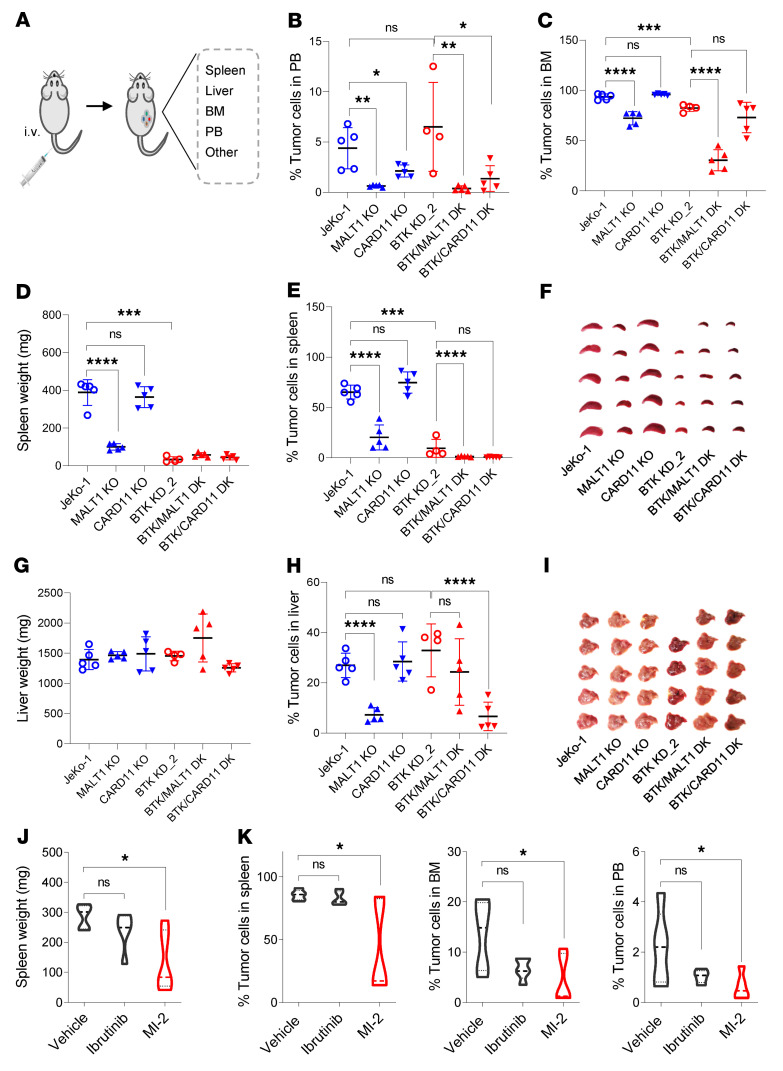
MALT1, but not CARD11, is critical for MCL cell dissemination to mouse spleen, liver, and BM. (**A**) Schematic illustration of disseminated cell line–derived CDX or PDX models. (**B**–**I**) Disseminated CDX models were established using JeKo-1 and JeKo BTK KD-2 cells, with or without *MALT1* or *CARD11* KO (*n* = 5 per group). At the end of the experiment, the spleen, liver, BM, and PB were harvested, imaged (**F** and **I**), and weighed (**D** and **G**) if appropriate. The tumor cell percentages in PB (**B**), BM (**C**), spleen (**E**), and liver (**H**) were determined by flow cytometry. The CD5^+^CD20^+^ cells represent tumor cell populations. (**J** and **K**) Freshly isolated primary PDX cells were injected intravenously into NSG mice to establish disseminated PDX models (*n* = 5 per group). At 6 weeks after injection, the mice were treated with vehicle, ibrutinib (50 mg/kg), or MI-2 (25 mg/kg) daily for 4 weeks. At the end of the experiment, mouse spleens were weighed (**J**). The CD5^+^CD20^+^ MCL cells were measured in spleens (**K**, left panel), BM (**K**, middle panel), and PB (**K**, right panel). One-way ANOVA was used in **B**–**E**, **H**, **J**, and **K**, where statistical significance was determined based on the adjusted *P* values using Šídák’s method. Data represent mean ± SD. *P < 0.05; **P < 0.01; ***P < 0.001; ****P < 0.0001.

We established disseminated CDX models by intravenous injection using JeKo-1 and JeKo BTK KD-2 cells with or without *MALT1* or *CARD11* KO (Figure 5A). In CDX models using JeKo-1 cells, the tumor cells accumulated markedly in the spleen, liver, BM, and even in PB (Figure 5, B–I), demonstrating MCL dissemination to these tissues. In JeKo-1 cells with *MALT1* KO, we observed a significant decrease in the frequency of tumor cells in the spleen (*P* < 0.0001), liver (*P* < 0.0001), BM (*P* < 0.0001), and PB (*P* < 0.01) (Figure 5, B–I). *BTK* KD in the JeKo-1 cells showed comparably decreased tumor cell presence in the spleen (*P* < 0.001) and BM (*P* < 0.001), which was also seen with *MALT1*-KO cells (Figure 5, C–F). However, *BTK* KD did not result in dramatic suppression in tumor cell presence in PB or liver (Figure 5, B and G–I).

*CARD11* KO in JeKo-1 cells showed modest effects on tumor cell presence in PB (*P* < 0.05), and it had no obvious impact on tumor cell incidence in the spleen, liver, or BM (Figure 5, B–G). *MALT1* KO in JeKo BTK KD-2 cells did not appear to have an effect on the tumor burden in the spleen or liver, since these cells were already very limited in their quantity at these sites. However, *MALT1* KO, but not *CARD11* KO, showed an additional significant decrease in the tumor cell incidence in BM (*P* < 0.0001) and PB (*P* < 0.01).

Next, we performed an in vivo short-term homing assay using a patient apheresis sample that contained 95% or greater MCL cells. The cells were pretreated with MI-2 or DMSO for 30 minutes, and then washed before injecting intravenously into NSG mice. The percentage of MCL cells in PB was determined to be similar for both groups at 1 hour after injection (Supplemental Figure 4A), but at 4 days after drug pretreatment it was significantly reduced in the MI-2 group compared with control in PB (*P* < 0.05), spleen (*P* < 0.05), and BM (*P* < 0.0001) (Supplemental Figure 4, B–D).

To assess the long-term effect of MALT1 inhibition on MCL tumor dissemination, we established PDX models by intravenous injection of IBN-R PDX cells. Treatment with MI-2, but not IBN, significantly decreased tumor burden in the spleen (*P* < 0.05), BM (*P* < 0.05), and PB (*P* < 0.05) (Figure 5, J and K). These data indicate that MALT1 inhibition is potentially useful in suppressing dissemination in IBN-R MCL tumors.

### MALT1 inhibition potently suppresses cell PI3K/AKT/mTOR signaling, adhesion, and migration in vitro.

**Figure 6:**
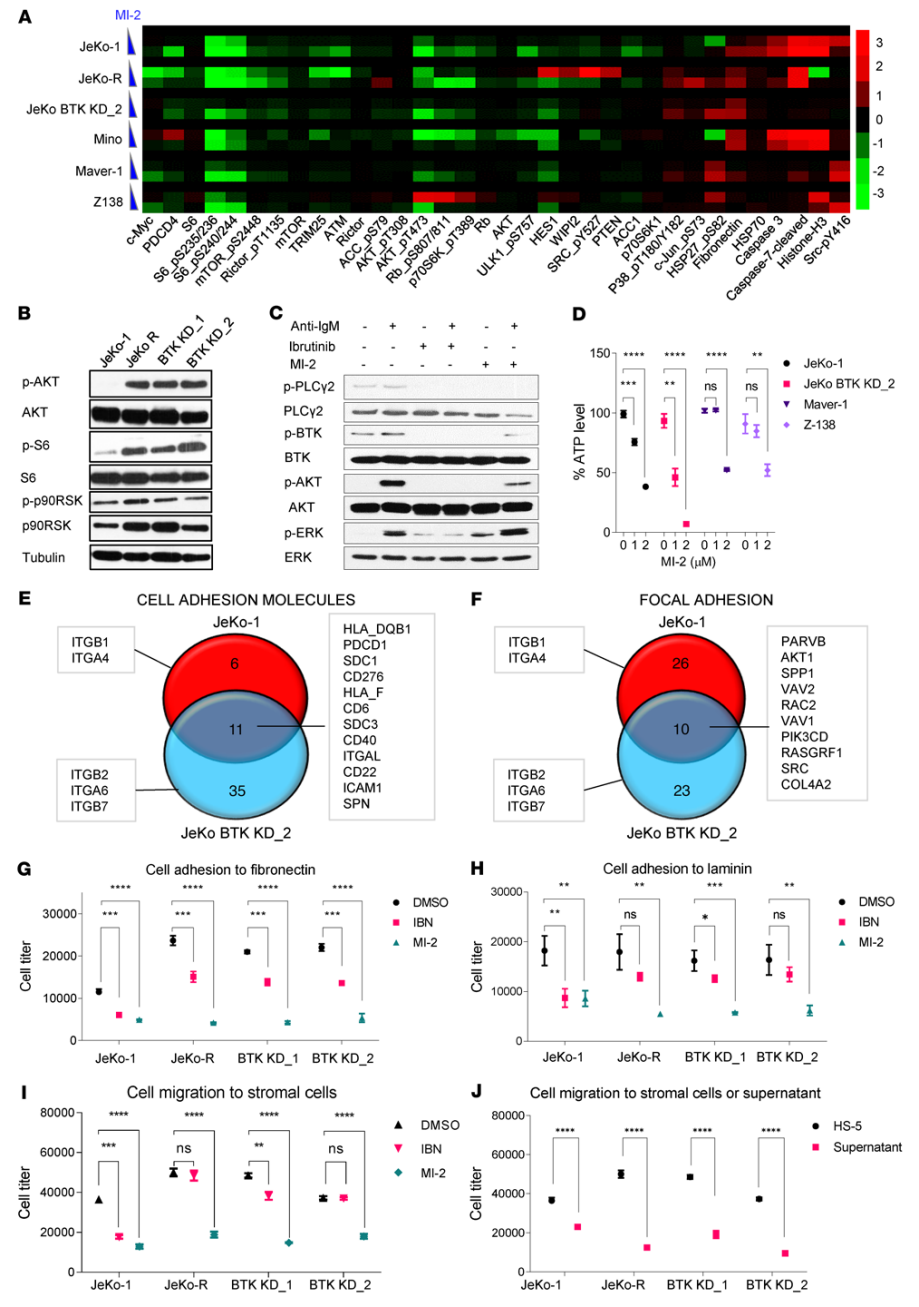
MALT1 inhibition leads to suppressed PI3K/AKT/mTOR signaling, cell adhesion, and migration in vitro. (**A**) RPPA analysis for MCL cell lines treated with MI-2 at 0, 1, or 2 μM for 6 hours. The heatmap shows the protein expression altered by MI-2 treatment. Each treatment for the indicated cell lines was set up in triplicate. (**B**) Phosphorylation of AKT, S6, and p90RSK was upregulated in JeKo-R and JeKo BTK KD-1 and -2 cells. (**C**) The phosphorylation of PLCγ2, BTK, AKT, and ERK was reduced upon MI-2 pretreatment followed by IgM stimulation in JeKo-1 cells. (**D**) ATP production was suppressed upon MI-2 treatment in MCL cells. Error bars were generated from 3 independent replicates. (**E** and **F**) The common DEGs involved in cell adhesion molecules and focal junction downregulated upon MI-2 treatment in both JeKo-1 (**E**) and JeKo BTK KD-2 (**F**) cells. Various integrin molecules altered upon MI-2 treatment are also indicated. (**G** and **H**) MCL cells were pretreated with DMSO, IBN at 5 μM, or MI-2 at 0.5 μM for 30 minutes and incubated in plates precoated with fibronectin (**G**) or laminin (**H**) for 4 hours. The cells adherent to fibronectin or laminin were measured and plotted. (**I**) MCL cells were pretreated with DMSO, IBN at 5 μM, or MI-2 at 1 μM for 30 minutes, added to Transwell inserts, and incubated in plates preseeded with a monolayer of stromal cells (HS-5) for 6 hours. The MCL cells that passed through the Transwell inserts were measured and plotted. (**J**) MCL cells were added in Transwell inserts and incubated in plates with preseeded (overnight) monolayers of stromal cells (HS-5) or with only the supernatants harvested from cultured stromal cells (overnight). The MCL cells that passed through the Transwell insert were measured and plotted. Error bars were generated from 3 independent replicates (**G**–**J**). Two-way ANOVA was used in **D** and **G**–**J**, and statistical significance was determined based on the adjusted *P* values using Šídák’s method. Data represent mean ± SD. *P < 0.05; **P < 0.01; ***P < 0.001; ****P < 0.0001.

We employed unbiased reverse-phase protein array (RPPA) analysis on cultured MCL cells to further understand the possible mechanisms of MALT1-driven dissemination. IBN-S and -R cells were treated with MI-2 for 6 hours and subjected to RPPA profiling. Many molecules involved in PI3K/AKT/mTOR signaling, including phosphorylated mTOR, rictor, AKT, P70-S6K, and S6, were dramatically downregulated in all of these cells following MI-2 treatment (Figure 6A).

Activation of PI3K/AKT/mTOR and integrin-β1 signaling is reportedly important for TME-driven IBN resistance (27) and was confirmed in IBN-R cells. Therefore, we hypothesized that MALT1 modulates MCL dissemination via regulating PI3K/AKT/mTOR and integrin-β1 signaling. Phosphorylation of AKT, S6, and p90RSK was upregulated in JeKo-1–derived IBN-R cell lines (Figure 6B). A 1-hour MI-2 pretreatment effectively blocked phosphorylation and activation of PLCγ2, BTK, and AKT when JeKo-1 cells were stimulated with anti-IgM for 5 minutes to induce BCR-triggered PI3K/AKT/mTOR signaling (Figure 6C). Consequently, MI-2 treatment also led to a dose-dependent reduction in intracellular ATP levels in all MCL cell lines tested (Figure 6D).

Our whole-transcriptome profiling revealed that apical junction regulation was one of the top cancer hallmarks notably downregulated following MALT1 inhibition (Supplemental Figure 3F). In addition to apical junction regulation, further analysis revealed that MALT1 inhibition suppressed multiple pathways involved in MCL dissemination, including cell adhesion molecules, focal adhesion complexes, and adherens junction proteins (Supplemental Figure 5A). DEG analysis showed that multiple integrin signaling molecules and cell adhesion molecules were downregulated upon MALT1 inhibition in both JeKo-1 and JeKo BTK KD-2 cells (Figure 6, E and F). Most of these proteins can directly or indirectly regulate integrin-mediated signaling and cell adhesion (37–39).

To address this further, we next screened extracellular matrix (ECM) components. Among 8 ECM components, we identified fibronectin and laminin as the dominant ECM factors involved in cell adhesion using MCL cell lines; interestingly, IBN-R cells (JeKo-R, JeKo BTK KD-1, and JeKo BTK KD-2) showed significantly higher cell adhesion to fibronectin (*P* < 0.0001), laminin (*P* < 0.0001), and bovine serum albumin (*P* < 0.0001) compared with IBN-S JeKo-1 cells (Supplemental Figure 5B). MI-2 treatment diminished MCL cell adhesion to fibronectin (*P* < 0.001), laminin (*P* < 0.01), and fetal bovine serum (FBS) (*P* < 0.0001), especially for IBN-R MCL cells (Figure 6, G and H, and Supplemental Figure 5C). Furthermore, MI-2 treatment diminished the capacity of MCL cells to migrate through Transwell inserts to a preseeded HS-5 stromal cell monolayer (*P* < 0.0001) (Figure 6I). MCL cells showed greater capacity in cell migration through Transwell inserts to stromal cells than to the culture supernatants harvested from HS-5 stromal cell cultures (*P* < 0.0001) (Figure 6J), suggesting that this HS-5–induced cell migration requires MCL–stromal cell contact. Together, these data suggest that MALT1 plays important roles in mediating cell adhesion, migration, and dissemination.

### Cotargeting of MALT1 and BTK overcomes IBN resistance in vitro and in vivo.

**Figure 7:**
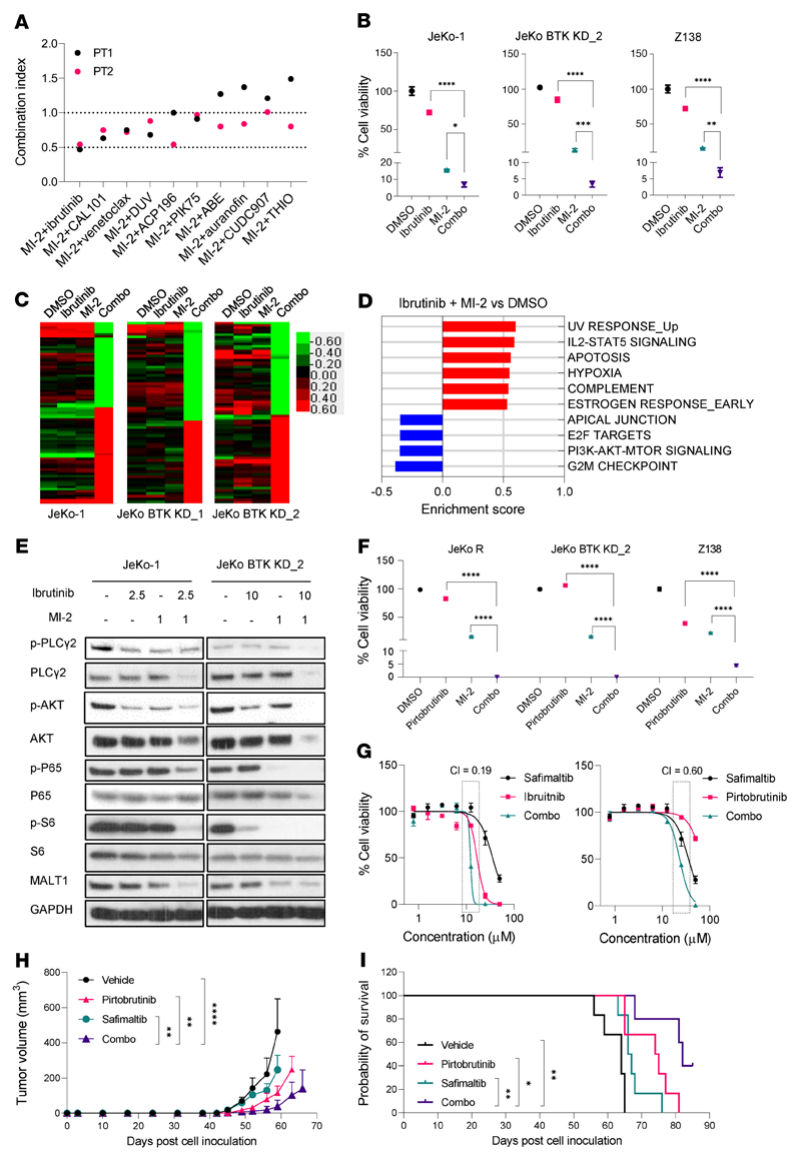
Dual targeting of BTK and MALT1 promotes potent anti-MCL activity in MCL cells with resistance to BTK inhibitors. (**A**) Combinational screen for MI-2 using 2 ibrutinib-resistant primary patient (PT) samples. The combination index was calculated and plotted for each combination. (**B**) MI-2 plus ibrutinib combination (combo) is synergistic against JeKo-1, JeKo BTK KD-2, and Z138 cells. (**C**) RPPA analysis for JeKo-1 and JeKo BTK KD-1 and -2 cells treated with MI-2 and ibrutinib, alone or in combination, for 6 hours. (**D**) GSEA revealed the top cancer hallmarks altered by the combination of MI-2 plus ibrutinib compared with DMSO control. Blue bars indicate the pathways downregulated by the ibrutinib plus MI-2 combination and red bars indicate the pathways upregulated by the combination. (**E**) Western blot analysis for JeKo-1 and JeKo BTK KD-2 cells treated with MI-2 and ibrutinib, alone or in combination, for 6 hours. (**F**) The combination effect of MI-2 plus pirtobrutinib combination ibrutinib-resistant JeKo-R, JeKo BTK KD-2, and Z138 cells. (**G**) The combination effect of safimaltib in combination with ibrutinib (left panel) or pirtobrutinib (right panel) in ibrutinib-resistant JeKo-R cells. The combination index is labeled for the indicated doses highlighted with the dotted rectangular boxes. Error bars were generated from 3 independent replicates (**B**, **F**, and **G**). (**H** and **I**) Freshly isolated primary PDX cells were injected subcutaneously into NSG mice to establish PDX models (*n* = 6 per group). When the subcutaneous tumors became palpable, the mice were treated with vehicle, pirtobrutinib (30 mg/kg twice daily), or safimaltib (50 mg/kg daily), alone or in combination. Tumor growth (**H**) and mouse survival (**I**) were monitored and plotted. Data represent mean ± SD. One-way ANOVA was used in **B** and **F**, 2-way ANOVA was used in **H**, and the pairwise log-rank test was used in **I**. Statistical significance was determined based on the adjusted *P* values using Šídák’s method. *P < 0.05; **P < 0.01; ***P < 0.001; ****P < 0.0001.

To screen for combinational therapies that have the potential to overcome IBN resistance, we treated MCL cells with MI-2 in combination with more than 10 drugs either FDA approved or under investigation. Among these, MI-2 in combination with IBN showed the greatest anti-MCL efficacy against 2 IBN-R patient samples and 1 IBN-R PDX sample (Figure 7A and Supplemental Figure 6A). This was further validated using MCL cell lines and additional patient and PDX samples (Figure 7B and Supplemental Figure 6B). Consistent with this, the MI-2 and IBN combination significantly induced apoptosis that was higher than with either single agent in JeKo-1 and JeKo BTK KD-1 and -2 cells (Supplemental Figure 6C).

Unbiased RPPA profiling showed that the MI-2 and IBN combination induced a synergistic effect on protein profiles of both JeKo-1 and JeKo BTK KD-2 cells (Figure 7C). The top cancer hallmarks that were suppressed by the combination were PI3K/AKT/mTOR signaling, apical junction proteins, G_2_/M checkpoint proteins, and E2F target proteins (Figure 7D), while apoptosis and hypoxia were among the top cancer hallmarks that were upregulated (Figure 7D). PI3K/AKT/mTOR and NF-κB signaling were further confirmed to be dramatically downregulated via Western blotting in JeKo-1 and JeKo BTK KD-2 cells treated with the MI-2 and IBN combination, compared with either single agent or vehicle (Figure 7E). MI-2 in combination with PBN also showed stronger antitumor activity than either single agent in IBN-R cells (*P* < 0.0001) (Figure 7F). Of note, JeKo-R and JeKo BTK KD-2 were demonstrated to be resistant to PBN (Supplemental Figure 6D).

MI-2 is commonly used as a chemical tool to inhibit MALT1 paracaspase activity in mechanistic and functional studies. For rational therapeutic development, we next tested another MALT1 inhibitor named safimaltib. Safimaltib was recently developed as a specific MALT1 paracaspase inhibitor (40), and it is currently under early clinical investigation in patients with non-Hodgkin lymphoma and chronic lymphocytic leukemia (ClinicalTrials.gov NCT03900598). Safimaltib showed effective anti-MCL activity not only in IBN-S cells, but also in both IBN-R and PBN-R cells (Supplemental Figure 6E). Additionally, safimaltib in combination with IBN or PBN was highly synergistic against JeKo-R cells in vitro (Figure 7G). Furthermore, safimaltib at 50 mg/kg daily in combination with PBN at 30 mg/kg twice daily dramatically inhibited tumor growth of an IBN-R PDX model (*P* < 0.01), and prolonged mouse survival (*P* < 0.05) beyond that observed by either single agent treatment (Figure 7, H and I). No effects on body weight were observed for either single agent or the combination treatment during this experiment (Supplemental Figure 6F). Together, these data indicate that cotargeting of MALT1 and BTK is promising to overcome resistance to BTK inhibitors in MCL.

## Discussion

We demonstrate that both MALT1 and CARD11 are crucial for MCL malignancy in IBN-S cells, and MALT1 becomes aberrantly expressed in IBN-R MCL cells and correlates negatively with CARD11 expression in these cells. Both MALT1 and CARD11 are crucial for the proliferation of IBN-S MCL cells in vitro and in vivo. However, only MALT1 is critical for the cell proliferation of IBN-R MCL cells and for MCL cell dissemination. These data suggest what we believe is a novel function for MALT1 in driving IBN resistance and in modulating MCL cell dissemination, both in a CARD11-independent manner.

*BTK* KD in JeKo-1 also demonstrated a defect in MCL cell dissemination to primary MCL sites like spleen and BM, but not to secondary sites like liver or PB. BTK can regulate integrin activation and cell adhesion via PI3K-dependent signaling (41). BTK inhibition by IBN has been shown to cause lymphocytosis, a process of compartment shifts of tumor cells from lymphoid tissues to the periphery, in patients with chronic lymphocytic leukemia (42) or MCL (43). Our unbiased transcriptomic profiling revealed that many genes involved in integrin signaling and cell adhesion were downregulated upon MALT1 inhibition in MCL cells. Furthermore, PI3KCD and AKT1, which are involved in PI3K/AKT/mTOR signaling, were also found to be downregulated upon MALT1 inhibition. Therefore, it is likely that, in addition to promoting cell survival through NF-κB signaling, MALT1 overexpression may enhance the capacity and scope of cell adhesion to prevent BTKi-induced lymphocytosis and tumor cell killing and thus confer BTKi resistance and immune evasion. Conversely, CARD11 seems to be dispensable in modulating MCL cell adhesion and dissemination. Our data suggest that an MCL-TME–mediated compensatory mechanism is critical for conferring BTKi resistance by integrin signaling and PI3K/AKT/mTOR signaling in a BTK- and MALT1-dependent manner.

MALT1 promotes T cell receptor–dependent activation of mTOR signaling and increases metabolism in CD4^+^ T cells (44), indicating crosstalk between NF-κB and mTOR signaling in regulating cellular metabolism. Our RPPA analysis showed that MI-2 treatment blocked AKT/mTOR signaling, which confers TME-driven IBN resistance in MCL cell lines (27). MI-2 treatment of MCL cells led to ROS production, loss of ΔΨ_m_, and thus abolished ATP production. These findings suggest that MALT1 plays a crucial role in regulating MCL cell survival and associated cellular energy metabolism via NF-κB and PI3K/AKT/mTOR signaling.

KO or pharmacological inhibition of MALT1 diminished MCL cell proliferation and survival irrespective of IBN sensitivity, suggesting that MALT1 is intimately associated with MCL viability. Recent clinical data on PBN (24) demonstrated that BTK is still targetable in many covalent BTKi–resistant patients. Therefore, dual targeting of MALT1 and BTK may greatly improve clinical outcomes in patients whether they are naive to BTKi therapy or have prior failure to it. Indeed, our preclinical data on dual targeting of MALT1 and BTK using combinations of the respective inhibitors demonstrates that this treatment strategy promotes potent anti-MCL activity in MCL cells with resistance to BTKis. In BTKi-sensitive cells, the dual targeting shuts down NF-κB and PI3K/AKT/mTOR signaling required for MCL cell survival, proliferation, adhesion, and dissemination. Conversely, in BTKi-resistant cells, MALT1 becomes aberrantly expressed and thus promotes cell survival and proliferation while conferring TME-mediated BTKi resistance as an alternative compensatory mechanism. Thus, dual targeting of BTK and MALT1 blocks multiple adaptive opportunities for the development of therapeutic resistance in MCL. Therefore, we envision that this can ultimately prevent therapy relapse and promote disease-free survival for MCL patients.

Safimaltib is a first-in-class, oral, specific MALT1 inhibitor currently under multiple clinical investigations to evaluate its clinical efficacy as single agent (ClinicalTrials.gov NCT03900598) or in combination with covalent BTKis IBN and JNJ-64264681 (ClinicalTrials.gov NCT04876092 and NCT04657224). Different from covalent BTK inhibitors, PBN is a noncovalent BTKi with high selectivity and potency. The BRUIN trial demonstrated that PBN can overcome resistance to a covalent BTKi in half of patients with prior failure to covalent BTKis (24). Therefore, we expect to see better efficacy of safimaltib in combination with PBN than with covalent BTKis. Our data provide evidence to support the potential clinical translation of safimaltib and PBN combination in patients with BTKi resistance in MCL and CLL.

In addition to MCL in this study, abnormal MALT1 activity is critical for driving MALT lymphoma. MALT lymphoma is the most common extranodal subtype of non-Hodgkin lymphoma. The chromosomal translocation t(11;18) (q21:q21) is characteristics of MALT lymphoma, which results in expression of an API2-MALT1 fusion oncoprotein. API2-MALT1 protein contains the N-terminus of API2 and the C-terminus of MALT1 (immunoglobulin-like domains and caspase-like domain) (45). API2-MALT1 can auto-oligomerize and thus has constitutive paracaspase activity, leading to potent and constitutive activation of NF-κB signaling (45). In such a way, it does not require any upstream signaling, including activation of CARD11 and assembly of the CARD11-BCL10-MALT1 complex, to mediate NF-κB signaling as the wild-type MALT1 does. In our study, we showed evidence that MALT1 is overexpressed and constitutively active in IBN-R cells. *MALT1* KO, but not *CARD11* KO, was able to dramatically diminish the tumor growth and dissemination in vitro and in vivo in the resistant MCL cells, supporting our notion that MALT1 hyperactivity critically contributes to IBN resistance via bypassing BTK/CARD11 upstream signaling. It has been shown that t(11;18)-positive lymphomas are associated with treatment resistance and a higher tendency to disseminate. Therefore, it is likely that MALT lymphoma is resistant to IBN treatment, which requires further investigation.

## Methods

### Cell samples.

MCL specimens and healthy PBMC samples were acquired from patients and healthy donors, respectively, after obtaining written informed consent. The MCL patient characteristics are presented in Supplemental Table 1.

### Reagents and antibodies.

IBN, PBN, MI-2, and VEN were purchased from Selleck Chemicals. Safimaltib was generated by custom synthesis through a contract research organization service. Supplemental Table 2 lists antibodies used and their sources. DMSO was purchased from Sigma-Aldrich.

### Cell culture.

The MCL cell lines JeKo-1, JeKo-R, JeKo BTK KD-1, JeKo BTK KD-2, Mino, Mino-VR, Rec-1, Rec-VR, Grant-519, Grant-519-VR, Maver-1, Z138, and SP-49 were maintained in RPMI 1640 medium supplemented with 1% penicillin/streptomycin, 25 mM 4-(2-hydroxyethyl)-1-piperazineethanesulfonic acid (HEPES), and 10% FBS (all from Sigma-Aldrich), and cultured in a CO_2_ incubator at 37°C as described previously (46). JeKo-1 cells are sensitive to IBN but resistant to VEN (Supplemental Figure 1A). JeKo BTK KD-1 and -2 cells derived from JeKo-1 cells have intrinsic IBN resistance due to BTK depletion (47), and JeKo-R cells have acquired resistance to IBN (47). Mino-VR, Rec-VR, and Granta-519-VR cells have acquired resistance to VEN (48). We grouped these cells into 3 pairings based on their drug sensitivity: (a) IBN-R versus IBN-S cells, (b) VEN-R versus VEN-S cells, and (c) Dual-R versus Dual-S cells (Supplemental Figure 1A). In addition, Maver-1 and Z138 are primarily resistant to IBN, while SP-49 is sensitive to IBN. Cell lines were authenticated by single-nucleotide polymorphism profile fingerprinting.

### Generation of MCL cells with stable gene KD via shRNA or with inducible expression of MALT1.

The method for generating MCL cells with stable *MALT1* KD has been described elsewhere (30). Quantitative real-time PCR was used to quantify the KD efficiency; 70% or more of mRNA expression was knocked down in all cells with stable *MALT1* KD.

### Generation of JeKo-MALT1 cells with inducible expression of MALT1.

MALT1 with C-terminal FLAG tag (MALT1-F) was subcloned into the pINDUCER lentiviral vector. Lentivirus carrying MALT1-F was prepared and used to transduce JeKo-1 cells as described previously (49). Doxycycline (1 μg/mL) was used to induce MALT1-F expression in JeKo-1 cells.

### Genetic KO of MALT1 or CARD11 in JeKo-1 and JeKo BTK KD-2 cells.

*MALT1*- and *CARD11*-KO cell lines were generated using the CRISPR/Cas9 genome editing system as described previously (47).

### Establishment of JeKo BTK KD GFP-Luc cells.

JeKo BTK KD-2 cells were infected with pseudolentiviruses packaged with pGF1-CMV-GFP-Luc and then sorted for GFP-positive cells. These cells were further expanded for a second sorting for GFP-positive cells and maintained in the same conditions as the other MCL cell lines.

### Bulk RNA sequencing and analysis.

Bulk RNA sequencing and analysis were performed as described previously (50). The sequencing data set has been deposited in the European Genome-Phenome Archive (EGA) database with the accession number EGAD00001009771.

### cDNA synthesis and quantitative real-time PCR.

The method for cDNA synthesis and quantitative real-time PCR has been described elsewhere (30). Briefly, first-strand cDNA was synthesized via the iScript cDNA Synthesis Kit (1725037, Bio-Rad) according to the manufacturer’s manual. Quantitative real-time PCR was conducted using SsoAdvanced Universal SYBR Green Supermix (1725271, Bio-Rad) according to the manufacturer’s manual. KiCqStart SYBR Green predesigned primers for *MALT1* and *GAPDH* (Sigma-Aldrich) were used in real-timer PCR experiments. *MALT1* mRNA expression was normalized to *GAPDH* and further normalized to one of the PBMCs samples from the healthy donors.

### Cell viability and apoptosis assays.

These assays were performed as described previously (46). Briefly, cells from MCL cell lines were seeded at 10,000 cells per well, and PDX tumor cells or primary patient tumor cells were seeded at 125,000 cells per well in a 96-well, white, flat-bottomed plate (3558, Corning). Cells were treated in triplicate with various doses of compounds. The compounds were prepared in DMSO to make a stock solution, from which 2-fold serial dilutions were prepared. Cell viability was determined at 72 hours (cell lines) or 24 hours (primary tumor cells) after seeding. For viability testing, cells to lysed with Cell Titer-Glo Luminescent Cell Viability Assay Reagent (Promega). Further, luminescence was measured via a BioTek Synergy HTX Multi-Mode microplate reader. For cell apoptosis assay, cells treated with or without MI-2 for 24 hours were stained with annexin V and propidium iodide (Abcam), followed by flow cytometry in a NovoCyte Flow Cytometer (ACEA Biosciences) to quantify apoptosis. The experiments were repeated at least 3 times.

### Immunoblotting.

The immunoblotting assay was performed as described previously (46). Briefly, 5 × 10^6^ to 10 × 10^6^ cells were seeded and treated as indicated. The cells were lysed in lysis buffer containing 50 mM HEPES (pH 7.4), 250 mM NaCl, 1 mM EDTA, 1% Nonidet P-40, 1 mM Na_3_VO_4_, 1 mM PMSF, 1 mM NaF, and a protease inhibitor mixture (all purchased from Roche Diagnostics). Protein concentration in cell lysates was measured using a Quick Start Bradford Protein Assay Kit (Bio-Rad) and the lysates were analyzed by SDS-PAGE and Western blotting. Primary antibodies used to detect total proteins, phosphorylated proteins, and their sources are presented in Supplemental Table 2.

### MALT1 ELISA.

The method for the MALT1 ELISA measuring MALT1 endogenous cleavage activity has been described elsewhere (51). Briefly, 0.2 × 10^6^ to 1 × 10^6^ MCL cells/mL were seeded on a 6-well plate and treated with MI-2 for 6 hours. The cells were harvested and analyzed by MALT1 ELISA to measure MALT1 endogenous cleavage activity. The experiments were repeated at least 3 times.

### NF-κB DNA-binding assay.

Nuclear fractions were extracted via the Nuclear Extract Kit (40410, ActiveMotif) and the DNA-binding activity of NF-κB family members was measured using a TransAM NF-κB Family Kit (43296, ActiveMotif) according to the manufacturer’s manual.

### RPPA analysis.

RPPA analysis was conducted as described previously (48). Briefly, RPPA analysis was conducted by the MD Anderson RPPA Core Facility. A total of 54 samples, representing 6 cell lines (JeKo-1, JeKo-R, JeKo BTK KD-2, Maver-1, Mino, and Z-138) and 3 doses of MI-2 (0, 1, 2 μM), tested in triplicate, were analyzed by RPPA. The slide images were quantified using MicroVigene 4.0 (Vigene-Tech). Spot-level raw data were processed using the R package SuperCurve, developed in-house. This package returns the estimated protein concentration (raw concentration), as well as a quality control score. Raw concentration data were normalized via median-centering each sample across all of the proteins to correct loading bias. In total, 307 antibodies and secondary antibody negative controls were analyzed. NormLog2_MedianCentered values were selected for heatmap generation. The differences in values of proteins in MI-2–treated samples were normalized to untreated samples, and differences less than –0.3 and greater than 0.3 in 3 or more cell lines were selected for the heatmap. The heatmap was generated using Cluster 3.0 software (https://cluster2.software.informer.com/3.0/) and visualized in Treeview (https://www.treeview.co.uk/). The results were presented in a high-resolution bitmap format. Each treatment was performed in triplicate.

### Measurement of cellular ROS, ΔΨ_m_, ATP, lactate, and glutamine.

The DCFDA/H2DCFDA - Cellular ROD Assay Kit (ab113851, Abcam), the ΔΨ_m_ assay kit (ab113852, Abcam), the ATP Colorimetric/Fluorometric Assay Kit (K354, Biovision), the Lactate Colorimetric Assay Kit II (K627-100, Biovision), and the Glutamine Colorimetric Assay Kit II (K556-100, Biovision) were used to detect cellular ROS levels, ΔΨ_m_, ATP levels, and extracellular lactate and glutamine, respectively, according to the corresponding manufacturers’ manuals. The experiments were repeated at least 3 times.

### Screen for ECM, cell adhesion, and migration assay.

These assays were performed as described previously (52). Briefly, the ECM Cell Adhesion Array Kit (ECM540, Millipore) was used to screen for MCL cell adhesion to the ECM according to the manufacturer’s manual. For specific cell adhesion assay, the plates were first coated with 10% FBS, fibronectin, or laminin, seeded with MCL cells pretreated with MI-2 at 0.5 μM for 30 minutes, and incubated for an additional 4 hours. The cells in suspension were thoroughly washed with PBS, and the resulting cells were lysed with Cell Titer-Glo Luminescent Cell Viability Assay Reagent (Promega). A BioTek Synergy HTX Multi-Mode microplate reader was used to quantify luminescence.

### Subcutaneous CDX or PDX models.

The in vivo experiments using subcutaneous CDX or PDX models were performed as described previously (48, 53). JeKo-1, JeKo-derived cells with BTK KD-2, *MALT1*-KO and/or *CARD11*-KO, JeKo BTK KD GFP-Luc cells, or PDX cells were injected subcutaneously (5 × 10^6^ cells per mouse) into 6- to 8-week-old NSG female mice (Jackson Laboratory). Drug treatment began once the tumor size became palpable. MI-2 was dissolved in 2% DMSO plus 30% PEG 300. The same vehicle without the drug served as the control. PBN was dissolved in 0.6% methylcellulose and 0.5% Tween 80. Safimaltib was dissolved in 5% DMSO plus 30% PEG 300 and 5% Tween 20. The mice were treated with vehicle, MI-2 (25 mg/kg, i.p., daily), safimaltib (50 mg/kg, orally, daily), PBN (30 mg/kg, orally, twice daily), alone or in combination. Mice were monitored daily for health condition and imaged weekly for tumor burden using the IVIS system (PerkinElmer) for the luciferase-expressing JeKo-BTK KD GFP-luc cells, or alternatively, the tumor size was weekly measured using calipers for non–luciferase-expressing models.

### In vivo short-term MCL cell homing experiment.

One MI-2–sensitive primary sample was stained with CellTracker Green CMFDA dye (Thermo Fisher Scientific) for 1 hour. The cells were washed and treated with DMSO or 1 μM MI-2 for 30 minutes. Cells were washed again and resuspended with complete medium, and then 2 × 10^7^ were intravenously injected into each NSG mouse (5 mice per group). After 4 days, the mice were euthanized and dissected to remove blood, spleen, and BM. CMFDA-positive cells representing MCL cells homing to each organ were detected by flow cytometry.

### In vivo disseminated CDX or PDX models.

The in vivo experiments using disseminated CDX or PDX models were performed as described previously (46). CDX or PDX models were established via the intravenous method. MCL cell lines or freshly isolated primary PDX cells (2 × 10^6^) were injected into NSG mice intravenously via the tail vein. The mice (*n* = 5 per group) were treated with vehicle, IBN (50 mg/kg, orally, daily), or MI-2 (25 mg/kg, i.p., daily) for 4 weeks at 6 weeks after injection. The mice were monitored daily for health condition and survival. At the end of the experiment, mice were euthanized and dissected for spleen, liver, blood, and BM. The spleen weight was measured, and the cells from blood, spleen, liver, and BM were isolated and stained with fluorophore-conjugated anti-CD5 and -CD20 antibodies. CD5 and CD20 double-positive cells representing MCL cells present in each organ or tissue were detected by flow cytometry.

### Statistics.

All experiments conducted in this study utilized at least 3 samples in each group. Using a 2-sided *t* test with a significance level of 0.05, each experiment had at least 80% power to detect an effect size of 3.1. For experiments that include more than 2 groups, 1-way ANOVA with at least 3 (or 5) samples in a group was able to detect at least an effect size of 1.36 (or 0.91), according to the significance level of 0.05 and study power of 80%. We found that the estimated effect size for the majority of our experiments was larger than the detectable limit, indicating the sufficiency of the sample sizes.

All analyses were descriptive in nature and performed using statistical software R v3.4.3 (https://cran.r-project.org/) with packages betareg v3.1-0, nlme v3.1-131, and survival v2.41-3, or GraphPad Prism v9. Where appropriate, descriptive statistics for each variable are presented as mean ± SD of the samples. The IC_50_ values were calculated from at least 3 independent experiments. Comparison of differences between 2 groups were conducted by 2-sided *t* test, or 2-sided Wilcoxon’s rank-sum test when the normality assumption did not hold. When multiple groups were involved, 1-way ANOVA was used. When 2 categorical independent variables (such as time and cell line) were considered, 2-way ANOVA was used. For endogenous MALT1 cleavage assays, statistical significance was calculated based on the *F* test for the slopes using a linear regression model. The log-rank test was used to assess the difference between groups in term of the progression-free survival. After demonstrating significance using ANOVA or in the presence of multiple-testing problems, the pairwise *P* values were adjusted using Šídák’s or Dunnett’s approach, depending on the analysis objective. The Benjamini-Hochberg method was applied to control the FDRs (*q* values). Results were considered statistically significant when *P* was less than 0.05: *P < 0.05, **P < 0.01, ***P < 0.001, ****P < 0.0001.

### Study approval.

Cell samples were acquired from patients and healthy donors after obtaining written informed consent following the University of Texas MD Anderson Cancer Center Institutional Review Board-approved protocols and in accordance with the Declaration of Helsinki. The Institutional Animal Care and Use Committee of The University of Texas MD Anderson Cancer Center approved the experimental protocols involving animals.

## Author contributions

MW and VCJ conceptualized, designed, and supervised the study, and acquired funding. VCJ, Y Liu, JL, SH, AJ, FY, JM, Y Li, YC, ZC, JV, MB, and JNB acquired data. VCJ, Y Liu, JL, SH, RL, QC, and MW analyzed data. VCJ, Y Liu, JL, SH, QC, HHL, WW, YY, LN, CRF, and MW interpreted data. VCJ wrote the original draft of the manuscript, which was reviewed and edited by VCJ and MW.

## Supplementary Material

Supplemental data

## Version 1 02/01/2023

Electronic publication

## Version 2 05/06/2025

Addition to Acknowledgments
