# Supplementary material for: Cotargeting of BTK and MALT1 overcomes resistance to BTK inhibitors in mantle cell lymphoma
Source: J Clin Invest. 2023 Feb 1;133(3):e165694. doi: 10.1172/JCI165694 (PMC9888382; doi:10.1172/JCI165694)
Supplement: Supplemental data [file jci-133-165694-s008.pdf]

Supplementary Data

Supplemental Tables

Supplementary Table S1. Clinical characteristics of patients with MCL.

Patients	Response to ibrutinib	Response to venetoclax	Sample collection time point	Sample source	% MCL	Supplementary Figure S2A
PT1	Progression	n.a.	8 days on ibrutinib	Apheresis	88%	PT1
PT2	Progression	n.a.	7 days on ibrutinib	Apheresis	97%	PT2
PT3	Progression	Progression	354 days on ibrutinib	Apheresis	92%	PT3
PT4	Progression	n.a.	25 days on ibrutinib	PB	98%	PT4
PT5	Progression	Progression	54 days before ibrutinib	PB	80%	PT5
PT6	progression	n.a.	1095 days on ibrutinib	Spleen	87%	PT6
PT7	Progression	Progression	1183 days before ibrutinib	Apheresis	99%	PT7
PT8	CR	n.a.	3 days before ibrutinib	Apheresis	93%	PT8
PT9	CR	n.a.	3 days before ibrutinib	Apheresis	96%	PT9
PT10	n.a.	n.a.	n.a.	Apheresis	98%	
PT11	CR	n.a.	4 days on acalabrutinib	Apheresis	90%	
PT12	CR	n.a.	0 day on ibrutinib	Apheresis	96%	
PT13	CR	n.a.	16 days before ibrutinib	PB	96%	
PT14	CR	n.a.	15 days before ibrutinib	BM	89%	
PT15	CR	n.a.	1 day on ibrutinib	Apheresis	96%	
PT16	CR	n.a.	1 day on ibrutinib	Apheresis	95%	
PT17	PR	n.a.	193 days before ibrutinib	Biopsy	87%	
PT18	CR	n.a.	0 day on ibrutinib	Apheresis	94%	
PT19	CR	n.a.	3 days before ibrutinib	Apheresis	93%	
PT20	CR	n.a.	4 days before ibrutinib	Apheresis	98%	
PT21	CR	n.a.	1 day before ibrutinib	PB	89%	
PT22	CR	n.a.	4 days before ibrutinib	Apheresis	98%	
PT23	Progression	Progression	1842 days on ibrutinib	Biopsy	80%	
PT24	Progression	n.a.	0 day on ibrutinib therapy	Pleural fluid	84%	

Supplementary Table S2. Antibodies used in this study.

Antibody	Cat. #	Company
AKT	4691	Cell Signaling Technology
BTK	3533	Cell Signaling Technology
CARD11	4435	Cell Signaling Technology
Caspase 3	9661	Cell Signaling Technology
CYLD	8462	Cell Signaling Technology
ERK	4695	Cell Signaling Technology
GAPDH	5174	Cell Signaling Technology
MALT1	2494	Cell Signaling Technology
p-AKT S473	4060	Cell Signaling Technology
p-ERK	4376	Cell Signaling Technology
p-p65	3036	Cell Signaling Technology
p-p90RSK	9346	Cell Signaling Technology
p-PLCg2 Y759	3874	Cell Signaling Technology
p-S6	8547	Cell Signaling Technology
p65	8242	Cell Signaling Technology
p90RSK	9355	Cell Signaling Technology
PARP	5623	Cell Signaling Technology
PLCg2	3872	Cell Signaling Technology
S6	2217	Cell Signaling Technology
BCL10	sc-9560	Santa Cruz Biotechnology
Rel B	sc-226	Santa Cruz Biotechnology
Tubulin	sc-8035	Santa Cruz Biotechnology
Flag	F3165	Sigma-Aldrich

Supplementary Figures

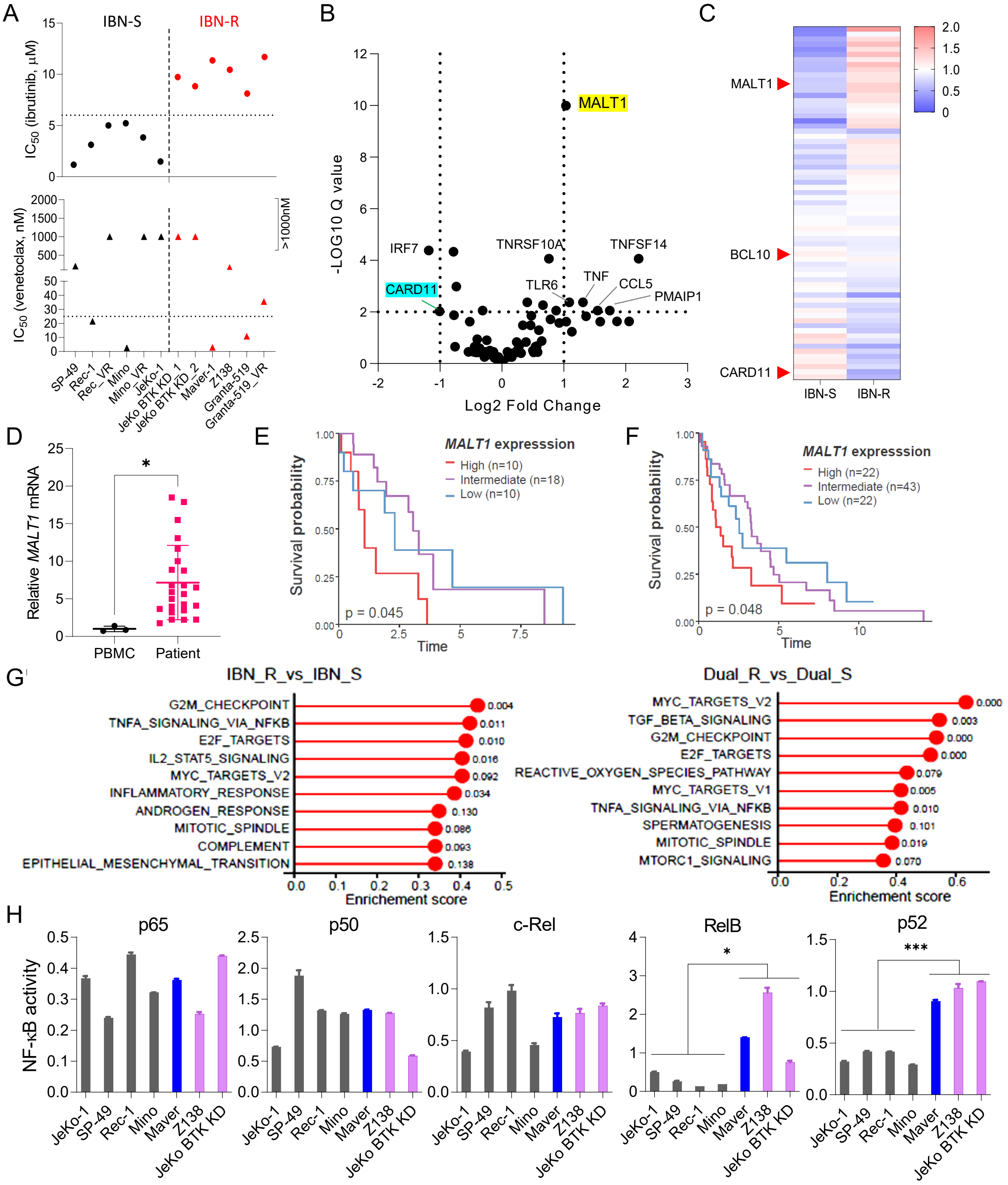

Supplementary Figure S1. MALT1 is overexpressed in ibrutinib-resistant MCL cell lines and primary MCL cells. (**A**) Dot plots show the IC_{50} values for ibrutinib (upper panel) and venetoclax (bottom panel) in 9 MCL cell lines. (**B-C**) Volcano plot (**B**) and heatmap (**C**) of DEGs involved in NF-κB signaling, comparing IBN-R vs IBN-S cells. (**D**) *MALT1* mRNA expression determined by quantitative PCR in MCL patients (n = 24) and PBMC samples (n = 3) from healthy donors (as controls). *MALT1* mRNA expression normalized to that of PBMC samples was shown. Statistical significance was calculated based on two-sided, two-sample t test. *, $p < 0.05$. (**E-F**) High *MALT1* mRNA expression correlated with poor patient outcomes using two patient cohorts published by other groups. Statistical significance was calculated by log rank test. (**G**) GSEA identified the top 10 cancer hallmarks upregulated in IBN-R cells compared to IBN-S cells (left) or in Dual-R cells compared to Dual-S cells (right). (**H**) NF-κB activity of all five NF-κB family members in each cell line. Error bars were generated from three independent replicates. Statistical significance was calculated based on two-sided, two-sample t test. *, $p < 0.05$; ***, $p < 0.001$.

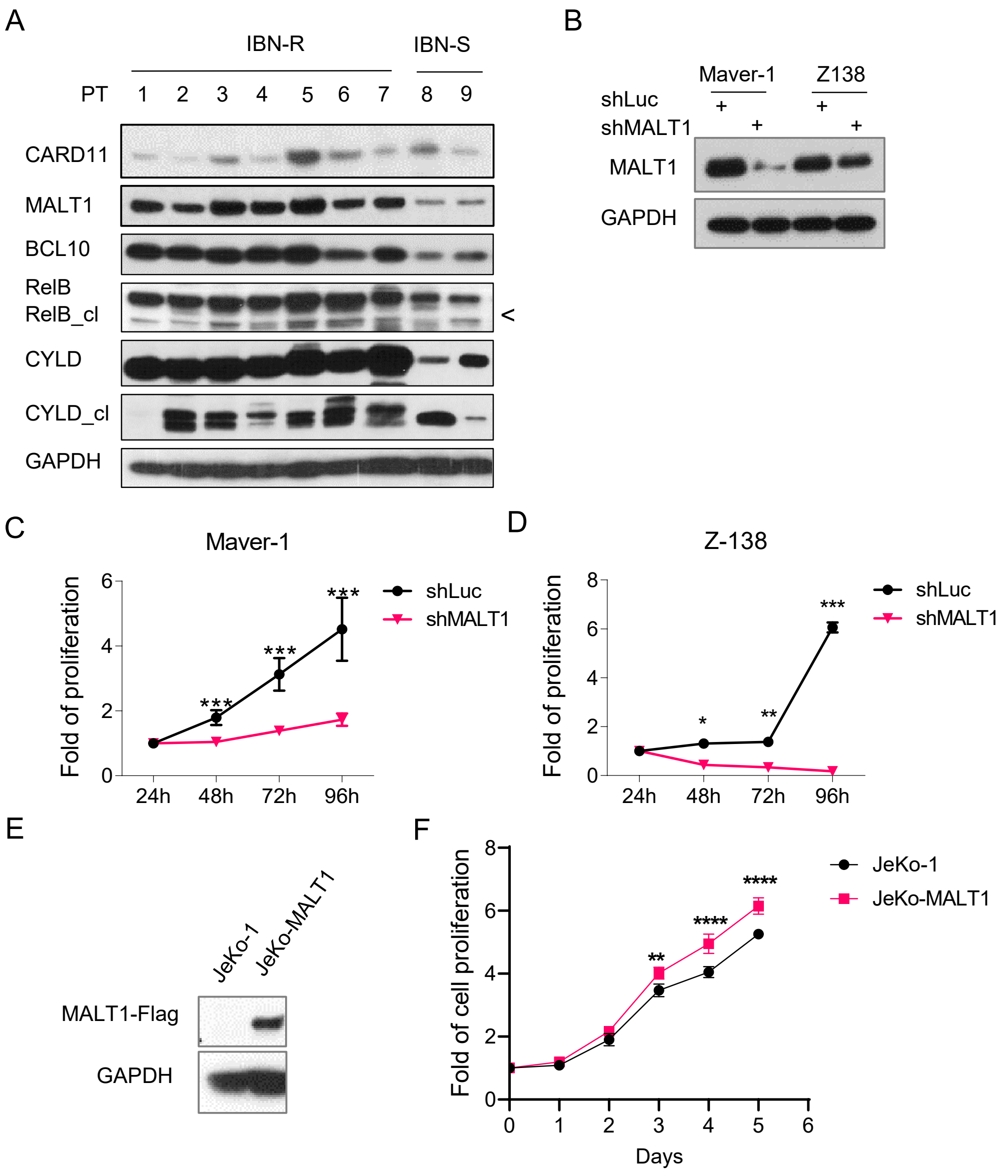

Supplementary Figure S2. MALT1 acts as an oncogenic tumor driver in ibrutinib-resistant MCL cells. (**A**) Expression of MALT1, CARD11, and BCL-10, and cleavage of MALT1 substrates, in IBN-R and IBN-S primary MCL cells. (**B-D**) MALT1 expression was detected by western blot in Maver-1 and Z138 with stable transduction of shLuc or shMALT1 (**B**). MALT1 knockdown resulted in suppression of cell proliferation in Maver-1 (**C**), and Z138 (**D**) cells. (**E-F**) JeKo-1 and JeKo-MALT1 cells were treated by doxycycline at 1 µg/ml for 24 hours and MALT1-Flag expression detected by anti-Flag antibody cells (**E**). Induced MALT1-Flag expression promoted cell proliferation of JeKo-MALT1 cells, compared to JeKo-1 cells (**F**). Error bars were generated from three independent replicates (**C, D and F**). Two-way ANOVA was used in (**C**), (**D**), and (**F**), and statistical significance was determined based on the adjusted p-values using the Šídák method. *, $p < 0.05$; **, $p < 0.01$; ***, $p < 0.001$; ****, $p < 0.0001$.

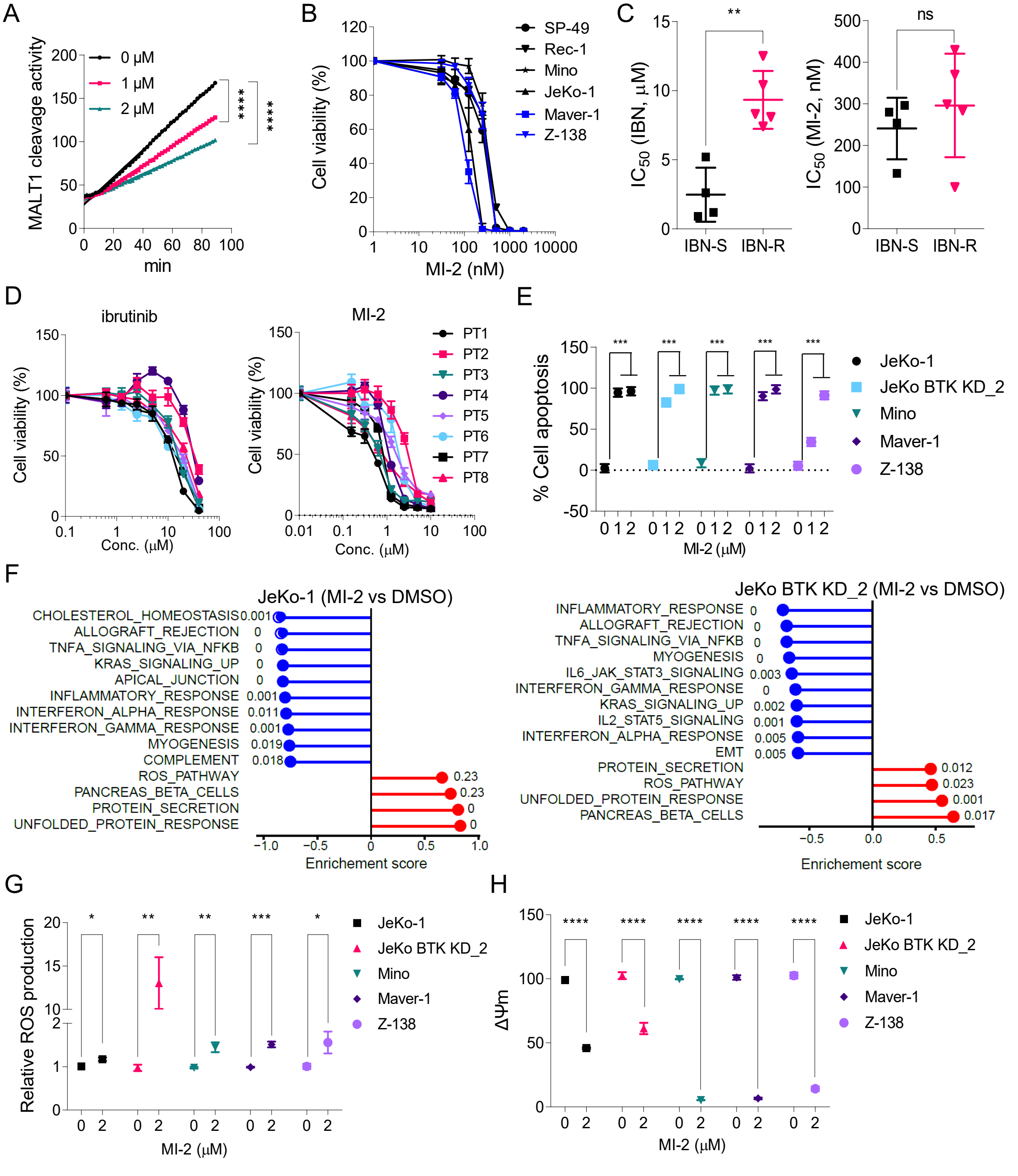

Supplementary Figure S3. MALT1 inhibition by MI-2 decreases MALT1 protease activity and suppresses cell growth in MCL. (**A**) Endogenous MALT1 cleavage activity detected in Mino cells upon MALT1 inhibition by MI-2 at the indicated concentration and treatment time. (**B**) MALT1 inhibitor MI-2 potently inhibited cell viability in MCL cells. (**C**) IC_{50} values of ibrutinib (left panel) and MI-2 (right panel) comparing IBN-R vs IBN-S cells. (**D**) Cell viability inhibited by ibrutinib or MI-2 in primary patient samples *ex vivo*. (**E**) MI-2 induced robust apoptosis in MCL cell lines. (**F**) GSEA analysis revealed that elevated ROS pathway was triggered upon MALT1 inhibition by MI-2 in JeKo-1 and JeKo BTK KD cells. Blue bars indicate negatively regulated pathways and red bars indicates positively regulated pathways. (**G-H**) ROS production (**G**) and ΔΨm (**H**) in JeKo-1, Mino, JeKo BTK KD_2, Maver-1, and Z-138 cells treated with MI-2 at 0 and 2 μM for 6 h (**G**) and 10 h (**H**). Error bars were generated from three independent replicates (**A, B, E, and G-H**). Two-way ANOVA was used in (**E**), (**G**), and (**H**), and statistical significance was determined based on the adjusted p-values using the Šídák method. ns, not significant ($p > 0.05$); *, $p < 0.05$; **, $p < 0.01$; ***, $p < 0.001$; ****, $p < 0.0001$.

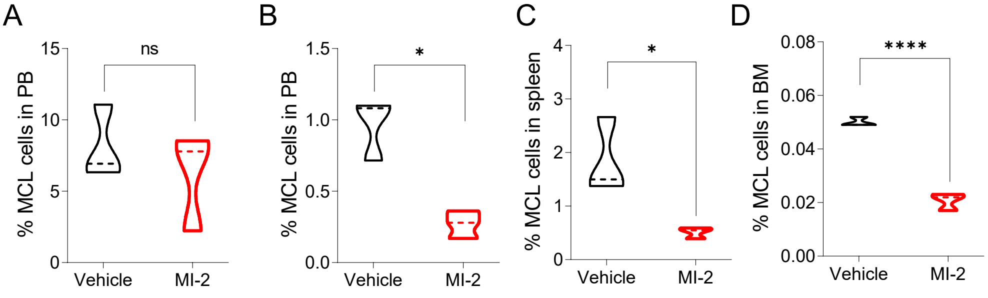

Supplementary Figure S4. MALT1 inhibition suppresses MCL cell dissemination in mouse peripheral blood (PB), spleen, and bone marrow (BM). (**A-D**) Primary MCL patient cells were stained with CellTracker Green CMFDA and pretreated with MI-2 at 1 µM for 30 min, then injected i.v. into NSG mice (n = 3 per group). $CD5^{+}CD20^{+}$ MCL cells were measured in PB (**A**) at 1 h post-injection, or in peripheral blood (**B**), spleen (**C**), and BM (**D**) at 4 d post-injection. Statistical significance was calculated based on two-sided, two-sample t test. ns, not significant ($p > 0.05$); *, $p < 0.05$; ****, $p < 0.0001$.

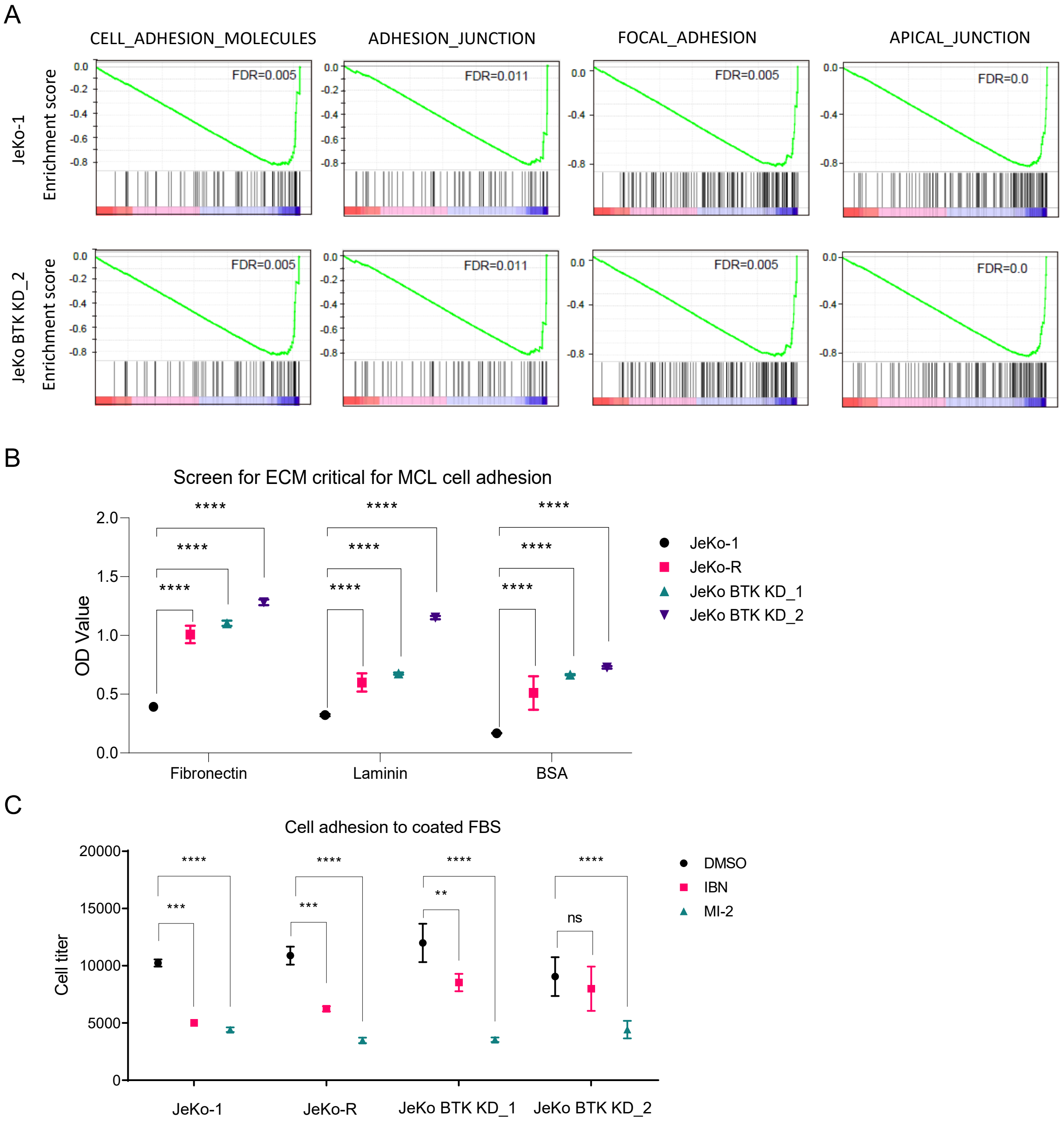

Supplementary Figure S5. MALT1 inhibition blocks MCL cell adhesion. (**A**) GSEA analysis revealed that cell adhesion molecules, adhesions junction, focal adhesion, and apical junction were significantly suppressed by MALT inhibition by MI-2 in JeKo-1 and JeKo BTK KD_2 cells. FDR (false discovery rate) was generated using the Benjamini–Hochberg method. (**B**) Screen for ECM using ECM array in JeKo-1, JeKo-R, JeKo BTK KD_1 and _2, and Mino cells. (**C**) JeKo-1, JeKo-R, JeKo BTK KD_1 and _2 were pretreated with DMSO, ibrutinib at 5 μM, or MI-2 at 0.5 μM for 30 min and incubated in plates pro-coated with fetal bovine serum (FBS) for 4 h. The cells adherent to FBS were measured and plotted. Error bars were generated from at least three independent replicates. Two-way ANOVA was used in (**B**) and (**C**), and statistical significance was determined based on the adjusted p-values using the Šídák method. ns, not significant ($p > 0.05$); **, $p < 0.01$; ***, $p < 0.001$; ****, $p < 0.0001$.

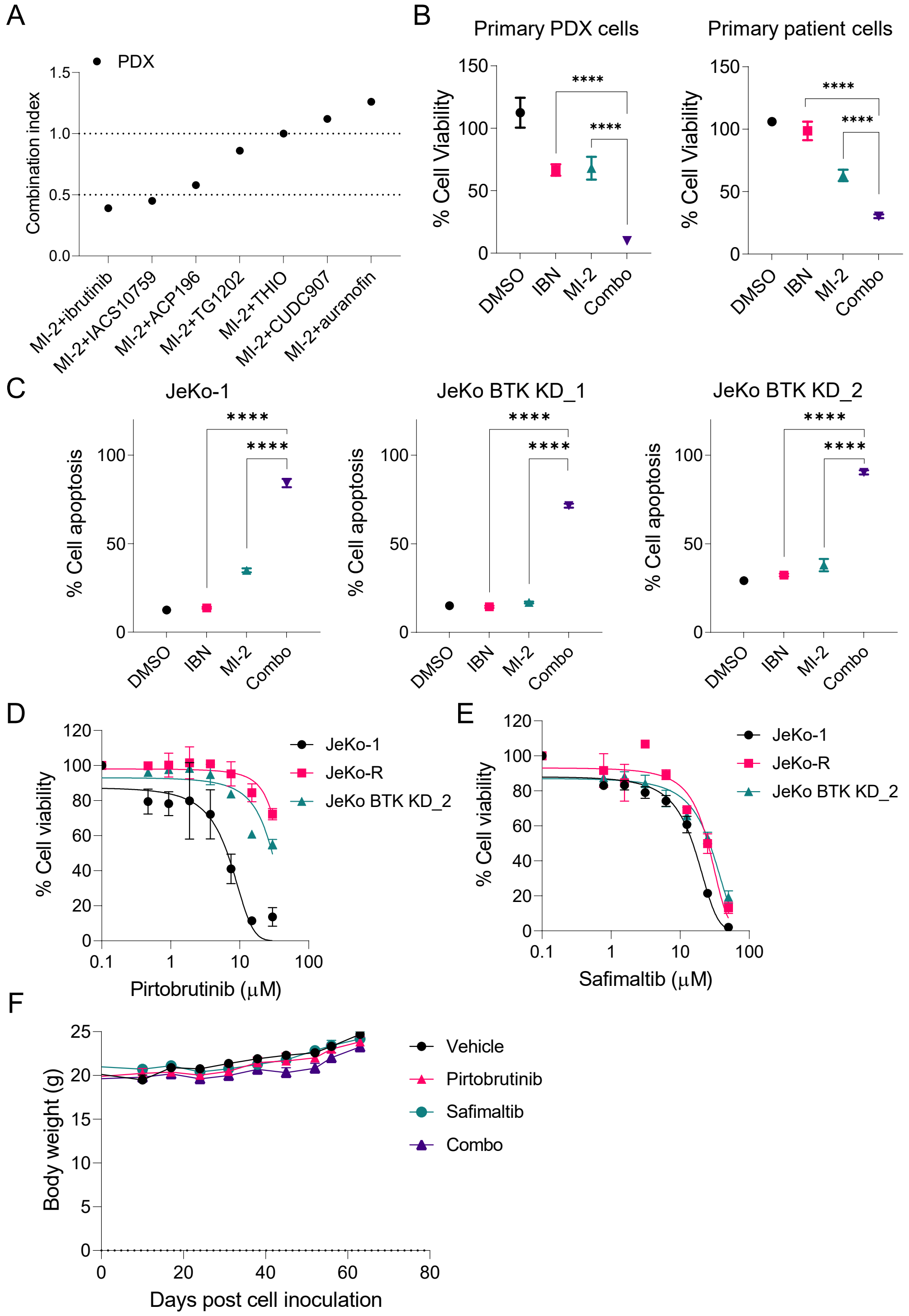

Supplementary Figure S6. Dual targeting of BTK and MALT1 promotes synthetic lethality in MCL cells. (**A**) Combinational screen for MI-2 using an ibrutinib-resistant primary PDX sample. The combination index was calculated and plotted for each combination. (**B**) MI-2 plus ibrutinib combination is synergistic against a primary PDX sample and a primary patient sample. (**C**) MI-2 plus ibrutinib combination is synergistic in inducing cell apoptosis in JeKo-1, JeKo BTK KD_1, and _2 cells. (**D-E**) The *in vitro* efficacy of pirtobrutinib (**D**) and safimaltib (**E**) in JeKo-1, JeKo-R, and JeKo BTK KD cells. Error bars were generated from three independent replicates (**B-D**). (**F**) Freshly isolated primary PDX cells were injected subcutaneously into NSG mice to establish PDX models (n = 6 per group). When the subcutaneous tumor became palpable, the mice were treated with vehicle, pirtobrutinib (30 mg/kg twice daily), or safimaltib (50 mg/kg daily), alone or in combination. Mouse body weights were monitored and plotted. One-way ANOVA was used in (**B**) and (**C**), and statistical significance was determined based on the adjusted p-values using the Šídák method. ****, $p < 0.0001$.
